# Exploration of Potential Genetic Biomarkers for Heart Failure: A Systematic Review

**DOI:** 10.3390/ijerph18115904

**Published:** 2021-05-31

**Authors:** Sek-Ying Chair, Judy-Yuet-Wa Chan, Mary-Miu-Yee Waye, Ting Liu, Bernard-Man-Hin Law, Wai-Tong Chien

**Affiliations:** 1 The Nethersole School of Nursing, Faculty of Medicine, The Chinese University of Hong Kong, Hong Kong, China; sychair@cuhk.edu.hk (S.-Y.C.); mary-waye@cuhk.edu.hk (M.-M.-Y.W.); tingliu@cuhk.edu.hk (T.L.); bernardlaw@cuhk.edu.hk (B.-M.-H.L.); wtchien@cuhk.edu.hk (W.-T.C.); 2 Asia-Pacific Genomic and Genetic Nursing Centre, The Nethersole School of Nursing, Faculty of Medicine, The Chinese University of Hong Kong, Hong Kong, China; 3 The Croucher Laboratory for Human Genomics, The Nethersole School of Nursing, Faculty of Medicine, The Chinese University of Hong Kong, Hong Kong, China

**Keywords:** heart failure, genetic biomarkers, single-nucleotide polymorphism, DNA methylation, transcriptome, microRNA, long noncoding RNA, circular RNA

## Abstract

Patients with heart failure (HF) often present with signs and symptoms that are often nonspecific and with a wide differential diagnosis, making diagnosis and prognosis of HF by clinical presentation alone challenging. Our knowledge on genetic diversity is rapidly evolving with high-throughput DNA sequencing technology, which makes a great potential for genetic biomarker development. The present review attempts to provide a comprehensive review on the modification of major genetic components in HF patients and to explore the potential application of these components as clinical biomarkers in the diagnosis and in monitoring the progress of HF. The literature search was conducted using six databases, resulting in the inclusion of eighteen studies in the review. The findings of these studies were summarized narratively. An appraisal of the reporting quality of the included studies was conducted using a twelve-item checklist adapted from the Strengthening the Reporting of Observational Studies in Epidemiology (STROBE) checklist. The findings showed that changes in genetic components in patients with HF compared to healthy controls could be noninvasive diagnostic or prognostic tools for HF with higher specificity and sensitivity in comparison with the traditional biomarkers. This review provided evidence for the potential of developing genetic biomarkers of HF.

## 1. Introduction

Heart failure (HF) remains a major cause of mortality, morbidity, hospitalizations, and poor quality of life. There is a global rise in the incidence of HF, with a regional variation in mortality. For example, Africa exhibits the highest case fatality rate of 34% for chronic HF, which is double the world average of 16.5% [1]. Given the increasing prevalence of HF worldwide, it is of utmost importance to ensure timely treatment and management of this potentially fatal condition among patients through various pharmacological and/or nonpharmacological means.

For the better prospect of timely management and/or treatment of HF and the optimal therapeutic effects of treatment among patients, accurate and early detection of the condition needs to be achieved. In the past, detection of HF relied largely on the presence of signs and symptoms of congestion, coupled with the examination of patients’ disease history [2]. However, this form of HF detection is prone to inaccuracies, particularly so among older adults because many of the characteristic signs and symptoms of HF are nonspecific, resulting in the suspicion of HF rather than giving a definitive diagnosis. This limits the utility of signs and symptoms in diagnosing HF [3,4]. Alternative ways for HF diagnosis need to be sought. It has been demonstrated that physiological changes often precede clinical deterioration that would lead to a patient attending hospital [5]. Invasive mechanisms such as pacemaker devices with physiological monitoring mechanisms can alert the physician to the clinical deterioration [6]. However, these are invasive and not all patients with HF have a pacemaker. Biomarkers, such as genes or other genetic materials found to be associated with the susceptibility of a disease, can serve as an alternative, noninvasive way for HF diagnosis. These biomarkers have become increasingly important in current medical practice as they offer an easy way to either diagnose an illness or to monitor disease progression. Indeed, biomarkers have been used to assess the status of a patient with HF along with the possibility of monitoring changes induced by patient management, primarily through the assessment of the level of expression of certain genes [7,8]. These biomarkers may, therefore, be used to assess whether therapies against HF are effective in treating the condition among patients. Consistent with this, biomarkers were suggested to have the potential to serve as indicators to the level of response to chemotherapeutic treatments and, thus, their treatment efficacies, thereby, facilitating the determination of the optimal chemotherapeutic drugs for use in treatment [9].

In addition, identification of biomarkers of HF may also potentially facilitate a better understanding of the etiologies of the disease. Indeed, previous studies had also suggested the potential of the identification of biomarkers in providing useful clues to the pathogenesis of neurodegenerative diseases, based on the discovery that variants of certain genes with known function may modify an individual’s susceptibility to these diseases [10]. Moreover, certain genes may potentially be differentially expressed in patients with various diseases including cardiovascular disease, compared to nondiseased controls [11]. Based on the known function of these genes, the molecular pathways that can contribute to the disease condition can be suggested. This not only could provide clues to further research into the disease etiologies but also provide novel molecular targets for the development of new therapies against the disease. Taken together, the identification of new biomarkers contributes to the facilitation of the diagnosis and treatment of diseases.

Given the value of biomarkers in informing us on the etiologies and diagnosis of diseases including HF, a review on the identified biomarkers of HF to date is needed. This would help provide updated, summarized data on the potential molecular pathways that can be targeted for therapeutic development against HF, as well as data on further choices of markers for assessing HF treatment efficacies. To the best of our knowledge, there are no recent systematic reviews that provide a summary of the identified biomarkers of HF. In this review, we aim to provide a comprehensive overview of the findings of previous studies examining the regulations in HF, so as to summarize the genetic biomarkers identified to date. Further, we want to explore the potential application of these findings in developing biomarkers for diagnosing and monitoring HF.

## 2. Materials and Methods

### 2.1. Search Strategy

The present systematic review was reported according to the criteria outlined in the Preferred Reporting Items for Systematic reviews and Meta-Analyses (PRISMA) guideline [12]. Online databases of PubMed, Ovid Medline, Ovid Nursing, Embase, CINAHL, and Cochrane were searched from inception to March 2021 to find articles that described the study of genetic modification in HF. We also performed manual searches of the bibliographies of relevant articles. The detailed search strategy used for the literature search is shown in Table 1.

### 2.2. Eligibility Criteria

A study was included in the review process if it (a) was written in English, (b) reported a case–control cohort study, (c) compared the differential expression of genetic markers in healthy subjects and patients with HF. Studies were excluded if cardiac diseases other than HF were analyzed. Moreover, articles that reported animal or cellular studies only, reviews, study protocols or conference abstracts were excluded. A full-text examination was performed to confirm their eligibility for inclusion or exclusion.

### 2.3. Data Extraction

After a literature search by a combination of keywords, the duplicates of records between different databases were screened and eliminated manually. Then, the selection of studies to be included in this review was performed by screening the titles and abstracts of the studies. Articles that did not meet the proposed selection criteria were excluded. The remaining articles were evaluated by full-text examination to confirm their eligibility for inclusion.

Further data extraction was performed by one author and verified by a second author after the inclusion of studies was confirmed. Extracted data included study design, study setting, applied country, cohort size, characteristics of participants, method of measurement of genetic change, and major findings of the studies. Any disagreements about data extraction from studies that occurred between the two authors were resolved by discussion with the third reviewer.

Owing to the diversity of outcome measures involved in the included studies, a meta-analysis of the extracted data was not feasible. The findings of the included studies have been presented in a narrative and tubular manner.

### 2.4. Quality Assessment

The methodological quality assessment was conducted using twelve selected items adopted from the Strengthening the Reporting of Observational Studies in Epidemiology (STROBE) checklist for cohort studies [13]. Selected items in the assessment include whether the included studies have provided an adequate description of the following: (1) the basic characteristics of the studies, including study design and settings, the rationale of the sample size used, and eligibility criteria used in subject recruitment; (2) the methodological details, including the variables, sources of measures used, statistical methods used, and how the authors address biases; (3) the outcomes of the studies, including the number of participants involved at each stage of the study, the demographic and clinical characteristics of participants, and the unadjusted and/or adjusted estimates obtained from these data. One mark was awarded for each item in the checklist if the item was achieved in the studies. Any items that were not achieved by the studies were given a zero score. Studies were categorized as low, moderate, or high quality if they scored <7, 7–9, or >9, respectively (arbitrary thresholds defined by authors).

## 3. Results

### 3.1. Search Results

**Figure 1:**
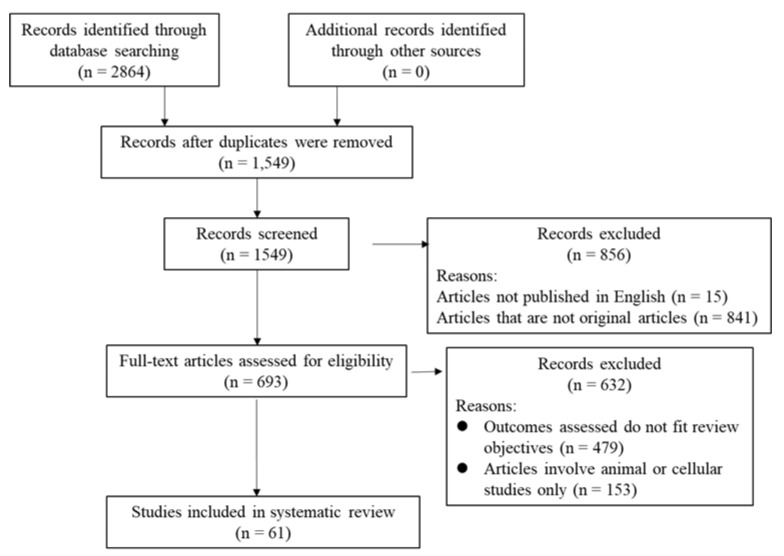
The PRISMA flow diagram.

The search strategy identified 2864 potentially relevant studies. Of these 2864 publications, 1315 were duplicates and were therefore excluded. The remaining 1549 records were subjected to title and abstract screening, among which, 15 articles were excluded as they were not published in English, and 841 were further excluded as they were not original articles. For the remaining 693 records, the full texts were retrieved, 632 of which were excluded for reasons including (1) outcomes assessed not fitting the review objectives or (2) articles not involving human subjects. Finally, 61 studies were eligible and included in this review. The PRISMA flow diagram showing the different stages of the study selection process is presented in Figure 1.

### 3.2. Quality of Included Studies

The ratings of the quality of the included studies are presented in Supplementary Table S1. Overall, the quality of the reporting of observational studies by the included studies was moderate to high. The scores of the included studies ranged from seven to eleven, and 83.6% of the included studies scored nine or above. All of the included studies described their outcomes and variables, the methods of measurement, and the number of participants at each stage well. Most of the included studies provided the characteristics of participants to show the clinical conditions of the participants. However, none of the studies described their rationale for sample size.

### 3.3. Characteristics of Included Studies

The characteristics of the included studies are listed in Supplementary Table S2. The included studies were published between 2007 and 2021. Twenty-three studies were conducted in China [14,15,16,17,18,19,20,21,22,23,24,25,26,27,28,29,30,31,32,33,34,35,36]; ten in the United States [37,38,39,40,41,42,43,44,45,46]; five in Iran [47,48,49,50,51]; four in Italy [52,53,54,55]; three in the Netherlands [56,57,58]; three in Germany [59,60,61]; two in Taiwan [62,63]; two in Australia [64,65]; and one each in New Zealand [66], Egypt [67], Japan [68], Czech Republic [69], Ireland [70], Hong Kong [71], the United Kingdom [72], Singapore [73], and Canada [74], respectively. All of the included studies are observational cohort studies and/or genome-wide association studies (GWASs). For the sample types, most of the studies involved peripheral blood and/or myocardial tissues, peripheral blood mononuclear cells (PBMCs), epicardial adipose tissue (EAT). The sample sizes, range between *n* = 4 and *n* = 977,323. The included studies assessed different genetic components. Twenty-four studies involved the assessment of single-nucleotide polymorphism (SNP) [18,19,20,21,26,28,33,39,40,42,43,45,47,48,49,50,51,52,62,63,67,69,72,74], eight studies were on the transcriptome [23,29,36,41,46,54,61,71], twenty-three studied microRNA (miRNA) [14,15,16,22,23,24,29,30,31,32,37,38,44,55,56,57,58,61,65,66,68,71,73], nine studied long noncoding RNA (lncRNA) [23,29,31,34,36,41,53,54,59], five were on DNA methylation [25,35,60,64,70], and two focused on circular RNA (circRNA) [17,27].

### 3.4. Reported Genetic Modifications in HF

#### 3.4.1. Transcriptome

Eight of the included studies reported the transcriptome analysis in HF [23,29,36,41,46,54,61,71]. Two studies involved the use of peripheral blood [29,71]. Five studies involved the use of cardiac tissues [23,41,46,54,61], and one study involved the use of EAT [36]. In one study, the result of the transcriptome analysis was validated in human peripheral blood [23]. These studies reported that the differential expressed genes were associated with various pathways including (1) fibrosis [23]; (2) cell adhesion, focal adhesion, cardiac muscle contraction [54]; (3) T cell receptor signaling pathway, primary immunodeficiency, endometrial cancer, drug metabolism cytochrome P450, tyrosine metabolism, complement and coagulation cascades, and Jak-STAT signaling pathway [36]; (4) mitochondrial oxidative signaling and noncanonical autophagy [46]. Hua et al. reported that all three RNA types, i.e., mRNAs, lncRNAs, and miRNAs, could largely distinguish HF patients from healthy controls. The results suggested that HF samples had distinct transcriptomic changes at multiple molecular levels when compared to control samples, implicating the potential use of transcriptomes as biomarkers for differentiating HF and normal controls [23]. This study identified that the genes *ASPN*, *COL1A1*, *COLQ*, and *IGFBP3* were associated with fibrosis. By investigating the survival time before heart transplantation, *COL1A1* was found as a potential biomarker for HF progression. A plasma level of *COL1A1* ≥ 256.5 ng/mL was associated with poor survival within 1 year of heart transplantation from HF (HR 7.4, 95% CI 3.5–15.8, Log-rank *p*-value <1.0 × 10^−4^) [23]. In the other two studies, right ventricle (RV)–specific myocardial biomarkers, which were related to HF, were reported [41,46]. Di Salvo et al. reported that the top 10 differential expressed genes (DEGs) exhibited high sensitivities, specificities and predictive values included *SERPINA3*, *SERPINA5*, *LCN6*, *LCN10*, *STEAP4*, *AKR1C1*, *STAC2*, *SPARCL1*, *VSIG4*, and *F8*. Among them, *STEAP4*, *SPARCL1*, *VSIG4* were differentially expressed in HF involving the RV versus HF involving the left ventricle (LV). The results supported that these three genes are potential RV-specific biomarkers [41]. Moreover, Tzimas et al. uncovered that a shift toward noncanonical autophagy in the failing RV was correlated with RV-specific *WIPI1* upregulation [46].

#### 3.4.2. LncRNA

Nine of the included studies reported the regulation of lncRNA in HF patients [23,29,31,34,36,41,53,54,59]. Four studies involved the examination of lncRNA in cardiac tissue, one study in EAT and one in PBMCs. In six studies, the modulation of lncRNA in HF patients was validated in peripheral blood [23,29,31,34,53,59]. In a study conducted by Greco et al. [53], 13 lncRNAs were significantly modulated (10 up- and 3 downregulated) in HF patients when compared to control subjects. In order to validate these identified HF lncRNA signatures, RNAs derived from more severe ischemic end-stage HF patients were analyzed. It was found that nine lncRNAs (*CDKN2B-AS1*, *EGOT*, *H19*, *HOTAIR*, *LOC285194*, *RMRP*, *RNY5*, *SOX2-OT*, and *SRA1*) were significantly modulated in a concordant manner in both end- and non-end stage HF patients. Further investigation using PBMCs from 25 HF patients and 18 healthy individuals revealed that *CDKN2B-AS1*, *HOTAIR*, and *LOC285194* showed similar modulation in PBMCs and heart tissue, suggesting the potential roles of these three genes as HF biomarkers [53]. In another study, the modulation of lncRNA heart-disease-associated transcript 2 (Heat2) was characterized in different HF cohorts, indicating its role as a potential biomarker [59]. Heat2 expression was elevated in HF patients with reduced ejection fraction (HFrEF) diagnosed with dilated cardiomyopathy (DCM) and ischemic cardiomyopathy (ICM), with *p* = 0.01 and *p* = 0.02, respectively. Furthermore, in a larger cohort study with HFrEF (*n* = 107), Heat2 was found to have a significant discriminatory power to predict the presence of HFrEF using whole-blood samples with an AUROC of 0.705 (95% CI: 0.604–0.805, *p* = 0.0004) [59]. Zhang et al. demonstrated that the expression of lncRNA taurine upregulated gene 1 (*TUG1*) is a potentially useful plasma biomarker for the diagnosis of heart failure with preserved ejection fraction (HFpEF). TUG1 was also confirmed to have a positive correlation with major serum marker N-terminal pro-brain natriuretic peptide (NT-proBNP) for HF [34].

#### 3.4.3. SNPs

Twenty-four of the included studies reported the association of polymorphisms of specific genes in HF [18,19,20,21,26,28,33,39,40,42,43,45,47,48,49,50,51,52,62,63,67,69,72,74]. These genes included *HDC* [18], *VDR* [19], *ADA* [20], *HRH3* [21], *IL-17A/IL-17RA* [26], i [28], *MHRT* [33], *HSPB7* and *FRMD4B* [39], *CLCNKA* [40], *TGFBR3* [42], *TNF-α* [47], *IL-4* [48], *IL-1* [49], *IL-2* [50], *IL-10* and *TGF-β1* [51], *KCNE1* [52], *AGTR1* [62], *MYBPC3* [63], *MIF* [67], *LEP* [69], *AGT* and *ACE* [74]. All of these studies involved the examination of SNPs in peripheral blood. When compared to other genetic modifications that involved the cardiac tissue, the number of samples examined in these studies was generally greater, from *n* = 150 [67] to *n* = 977,323 [72]. All of these studies have reported the allelic/genotype frequency of the SNPs under investigation. Six of them also showed the multivariate analysis for SNPs and the risk of HF [18,20,21,26,28,52]. Among the included studies, seventeen studies showed the results of haplotype distribution analysis [18,19,20,21,26,28,33,47,48,49,50,51,62,63,67,69,74]. In He et al., the clinical associations of polymorphisms in *HRH2*, *HRH3*, *DAO*, and *HNMT* with chronic heart failure (CHF) were investigated [21]. By genotyping 11 SNPs in the blood of 333 CHF patients and 354 normal controls, it was found that the HRH3 rs3787429 polymorphism was associated with CHF risk (*p* < 0.001). The T allele of rs3787429 was more frequent in the control group than in the CHF group and exhibited a protective effect against CHF (adjusted odds ratio, 0.608; 95% CI, 0.470–0.786; *p* < 0.01) [21]. These data further strengthen the independent role of rs3787429 polymorphism in modulating CHF susceptibility. In the included studies, the genetic polymorphism of the *KCNE1* gene was reported to be associated with HF predisposition in two study populations [52]. In HF patients from Florence, Italy, a higher prevalence of the *KCNE1* S38G variant was associated with a significant predisposition to HF under genetic models of inheritance (*p* < 0.05). In order to validate the results, the study was replicated in another cohort in Pisa. Similar findings were demonstrated concerning the role of the *KCNE1* S38G polymorphism in modulating susceptibility to HF [52]. Genetic polymorphism of the *AGTR1* gene had been studied and rs16860760, rs389566, as well as rs5186 were found to be significantly associated with diastolic HF (*p* = 0.004, 0.002 and 0.002, respectively) [62]. *ErbB4* gene polymorphisms were associated with the risk, severity, and prognosis of congestive HF [28]. Thirteen SNPs of *NRG-1/ErbB2/ErbB4* genes were analyzed between HF patients and control subjects. In the *ErbB4* gene, the variants rs10932374 and rs1595064 were associated with reduced risk of HF (*p* = 0.039 and 0.007, respectively) while the variants rs13003941 and rs1595065 were associated with increased risk of HF (*p* = 0.015 and 0.005, respectively). The T variant of rs13003941 was associated with a larger left ventricle (*p* = 0.048) and increased risk of overall death (RR 1.48, 95% CI 1.01–2.18, *p* = 0.045) and cardiovascular death (RR 1.56, 95% CI 1.04–2.33, *p* = 0.03) [28]. Elevated cytokine levels have been revealed in patients with HF. Mahmoudi et al. have carried out studies in Iran and reported the association of *IL-1*, *IL-2*, *IL-4*, *IL-6*, *IL-10*, *TNF-α* and *TGF-β1* gene polymorphism with HF [47,48,49,50,51]. The association of *IL-17A* and *IL-17RA* polymorphism with the risk of congestive HF and its mortality rate is also reported [26]. Three studies involved GWAS [42,45,72]. Kao et al. found that SNP of *TGFBR3* rs6996224 was significantly associated with HFpEF (OR = 11.3, *p* = 3.70 × 10^−9^) [42]. Schneider et al. validated two SNPs obtained from GWAS and revealed that one SNP, rs28714259, was associated with increased CHF risk (*p* = 0.04, OR = 1.9) and with a decreased left ventricular ejection fraction (*p* = 0.018, OR = 4.2) [45]. Shah et al. conducted a GWAS comprising 26 studies and finally found that 12 variants at 11 genomic loci were associated with HF. All of them showed association with either coronary artery disease (CAD), atrial fibrillation (AF), or reduced left ventricular function while non-CAD-associated loci implicate genes that are involved in cardiac development, protein homoeostasis, and cellular senescence [72].

#### 3.4.4. miRNA

Twenty-three of the included studies reported the changes of miRNA in HF [14,15,16,22,23,24,29,30,31,32,37,38,44,55,56,57,58,61,65,66,68,71,73]. All of them examined the outcome in peripheral blood except one, which examined the miRNA changes in cardiac tissue only [61]. Three of them examined the outcome in cardiac tissue and validated the results in peripheral blood [23,24,37]. Two studies have reported the correlation of miRNA changes with clinical outcomes in HF [30,58]. Akat et al. reported that myomiRs displayed the biggest differences in levels among advanced HF patients compared with normal subjects [37]. The cardiac-specific myomiRs, mir-208b(1), mir-208a(1), and mir-499(1), and the muscle-specific mir-1-1(4) and mir-133b(2) were 143-, 78-, 28-, 18-, and 21-fold higher in advanced HF at left ventricular assist device (LVAD) implantation compared with healthy controls [37]. The miRNA changes in advanced HF reversed 3 and 6 months after LVAD implantation. The levels of the myomiRs mir-208a(1), mir-208b(1), mir-499(1), and mir-1-1(4) dropped as early as 3 months after the initiation of LVAD support, approaching normal levels [37]. Heart-specific myomiRs (mir-208a, mir-208b, and mir-499) also performed similarly with *cardiac troponin I*, an established biomarker for heart injury [37]. In a study conducted by Wu et al., the expression levels of three microRNAs, miR-92b-5p, miR-192-5p, and miR-320a, were tested. Only miR-92b-5p was found to be enriched in HF patients with HFrEF when compared to control subjects (*p* < 0.001) [30]. The analysis also confirmed its ability to discriminate HFrEF from healthy controls with a sensitivity of 71.4% and a specificity of 83.3%. The results demonstrated that miR-92b-5p may serve as a marker for HFrEF diagnosis [30]. Two other studies also identified potential miRNA markers for HFrEF discrimination from normal control. These included miR-3135b and miR-3908 by Chen et al. [14] and miR-125a-5p, miR-183-3p, miR-193b-3p, miR-211-5p, miR-494, and miR-638 by Wong et al. [73]. In another study, 752 miRNAs were screened in moderate and advanced HF patients. Five miRNAs including miR-26a-5p, miR-145-3p, miR-150-5p, miR-485-3p, and miR-487b-3p were found to be significantly dysregulated in HF [55]. In the validation study by comparing advanced HF patients to healthy subjects and moderate HF patients, only miR-26a-5p and miR-150-5p were confirmed to be downregulated. Further analysis demonstrated the association of miR-150-5p with HF severity [55]. In the study conducted by Vegter et al. [58], the association between acute HF-specific circulating miRNAs and well-known HF biomarkers were identified. The results showed that seven miRNAs were negatively correlated to biomarkers indicative for a worse clinical outcome in HF patient group [58]. Correlations were found between miR-16-5p and CRP (R = −0.66, *p* = 0.0027), miR-106a-5p and creatinine (R = −0.68, *p* = 0.002), miR-223-3p and GDF-15 (R = −0.69, *p* = 0.0015), miR-652-3p and sST-2 (R = −0.77, *p* < 0.001), miR-199a-3p and PCT (R = −0.72, *p* < 0.001), miR-18a-5p and PCT (R = −0.68, *p* = 0.002), and miR-199a-3p and galectin-3 (R = −0.73, *p* < 0.001) [58]. These correlations were significant to those patients with the worst clinical course during hospitalization, which was characterized by a worsening renal function, the need for rescue therapy, worsening dyspnea symptoms at 24 to 48 h and a poor outcome.

#### 3.4.5. DNA Methylation

Five of the included studies reported the change of DNA methylation in HF [25,35,60,64,70]. All of them examined the DNA methylation pattern using peripheral blood. In one study, the results were confirmed in both cardiac tissue and peripheral blood [60]. Meder et al. reported the results when the methylation pattern of myocardial tissue and peripheral blood was compared. It was found that three epigenetic loci significantly overlapped between the tissue and blood (OR: 28; *p* < 0.001). The resolved genes were *B9 protein domain 1* (*B9D1*) (hypomethylated), *doublecortin-like kinase 2* (hypomethylated) and *neurotrimin* (hypermethylated). Among these three genes, *B9D1* has the greatest potential to serve as a biomarker for HF because it was found to have an area under the curve of >87% in the peripheral blood discovery cohort with robust replication in myocardial tissue as well as in peripheral blood verification cohorts [60]. In the other four studies, various potential epigenetic biomarkers were reported. They include *SLC2A1*, *MPV17L* and *PLEC* [25]; *METTL3*, *METTL4*, *KIAA1429*, *FTO* and *YTHDF2* [35]; *HDAC9* [64]; *HEY2*, *MSR1*, *MYOM3*, *COX17*, *MMP2*, *CTGF*, miR-24-1, and miR-155 [70].

#### 3.4.6. circRNA

Two of the included studies reported the results on circRNA [17,27]. Both studies involved samples taken from peripheral blood. Han et al. analyzed blood samples from HF patients and healthy controls by using next-generation sequencing (NGS) to screen for differentially expressed circRNAs. Six differentially expressed circRNAs were then validated by real-time PCR (RT-PCR) and hsa_circ_0097435 expression was confirmed to be significantly upregulated in HF patients [17]. In another study, Sun et al. reported 696 differentially expressed circRNAs among HF patients and controls [27]. Verification by RT-PCR showed that hsa_circ_0112085 (*p* = 0.0032), hsa_circ_0062960 (*p* = 0.0006), hsa_circ_0053919 (*p* = 0.0074), and hsa_circ_0014010 (*p* = 0.025) had significantly higher expression in patients with HF. Correlation analysis showed that the expression of hsa_circ_0062960 was highly correlated with B-type natriuretic peptide (BNP) serum levels. The area under the ROC curve (AUC) of hsa_circ_0062960, hsa_circ_0112085, and hsa_circ_0053919 for HF diagnosis was 0.838 ((0.740–0.937), *p* < 0.0001), 0.817 ((0.713–0.921), *p* < 0.0001), and 0.759 ((0.631–0.887), *p* = 0.001), respectively. The serum BNP level was strongly correlated with the expression of hsa_circ_0062960 (R = 0.649, *p* = 0.003) [27].

## 4. Discussion

The findings of the present review demonstrate the genetic regulations in HF and their roles in pathogenic processes, which allow the potential development of biomarkers for the diagnosis and prognosis of HF (Table 2). The review identified six genomic components that are modulated in HF including transcriptome, miRNA, SNP, lncRNA, DNA methylation, and circRNA. Due to the heterogeneity of the studies, it is difficult to make a direct comparison between the included studies. In another way, the advantage and feasibility of exploring the findings in clinical applications will be discussed.

Biomarkers can aid in understanding the prediction, cause, diagnosis, progression, regression, or outcome of treatment of disease. It could be the cellular, biochemical, or molecular alterations that are measurable in biological media such as human tissues, cells, or fluids [75]. The World Health Organization (WHO) has defined a biomarker as any substance, structure, or process that can be measured and can predict the incidence of an outcome, a disease or the effects of treatments, interventions, and unintended environmental exposures [76]. BNP and NT-proBNP are currently widely used biomarkers for HF. They possess some qualities including the following: (1) they are conveniently measured and easily interpretable; (2) they can confirm or exclude a diagnosis of HF; (3) they can predict which patients are at risk of developing HF; (4) they provide prognostic information for those with a diagnosis of HF; (5) they guide therapy in HF; (6) they serve as surrogate endpoints in clinical trials [77]. In this review, some of the included studies have compared the performance of their findings to established HF biomarkers [27,30,37,55,57,58,68,73]. Five studies analyzed the correlation of their findings with BNP [27,68] and NT-proBNP [55,57,73]. The findings in Akat et al. demonstrated that heart-specific myomiRs miRNA (mir-208a, mir-208b, and mir-499) performed similarly with cardiac troponin I, an established biomarker for heart injury [37]. In the study by Wu et al., the sensitivity and specificity of exo-miR-92b-5p as a biomarker were analyzed to confirm its ability to discriminate patients with HFrEF from healthy controls. A sensitivity of 71.4% and a specificity of 83.3% were achieved [30]. In the study conducted by Vegter et al., the association between acute HF-specific circulating miRNAs and well-known HF biomarkers was also identified [58].

Complex diseases are mediated via genomic dysregulation in multiple tissues. However, invasive procedures are required to obtain most tissues. On the other side, whole blood is a fast, simple, and safe source for genomic studies. In this review, both tissue-derived and blood-derived biomarkers were included. It has been reported that an individual’s whole-blood transcriptome can significantly predict tissue-specific expression levels for ~60% of the genes on average across 32 tissues, with up to 81% of the genes in skeletal muscle [78]. In the future, blood transcriptome can help detect biomarkers in some cases and to be more accurate and effective model to predict tissue-specific expression. In six of the included studies, they were included in both the screening phase and the validation phase [14,17,22,23,24,27,30,37,53,55,56,59,66,73]. The genetic modulation profile was firstly screened in cardiac tissue [23,24,37,53] or in peripheral blood [14,17,22,27,30,55,56,59,66,73]. Then, specific genetic changes were validated in peripheral blood (with the same or different cohorts). It should be noted that these studies with screening and validation phases involved the assessment of miRNA [14,22,23,24,30,37,55,56,66,73], lncRNA [23,53,59], mRNA [23], and circRNA [17,27] but not SNP. Since the SNP of a single gene may include many allelic variants, the complex variations in SNP make it complicated as a screening tool. However, from the findings of this review, it was observed that SNP detection of specific genes could be done in large cohorts. The sample size of two of the included studies assessing SNP involved >1000 subjects [26,28].

Novel biomarkers may supplement traditional clinical and laboratory testing to improve understanding of the complex disease process of HF and possibly serve to personalize care for those affected through better individual phenotyping. In one of the included studies, RNA-seq was used to generate the transcriptional profile of end-stage human HF right ventricular myocardium and unused donor RV myocardium to identify candidate RV myocardial biomarkers. The transcriptomes of HF RVs were reviewed with echocardiographic dysfunction versus echocardiographic normal function to identify candidate biomarkers of RV dysfunction in end-stage HF. Finally, RV-specific biomarkers (*STEAP4*, *SPARCL1*, and *VSIG4*) were identified by comparing the findings between RV and LV transcriptomes [31]. The other two included studies, respectively, found Ex-miR-92b-5p and *Heat2* to be potential biomarkers for diagnosing HFrEF [30,59]. Studies also identified potential biomarkers for diagnosis or prognosis of left ventricular dysfunction [54] and right ventricular dysfunction [41,46].

Apart from diagnostic biomarkers, the findings in four included studies demonstrated the potential of biomarkers for prognosis [21,26,28,33,36,37,41,58,61,62]. The association of *IL17RA* polymorphism with mortality [26] and association of *ErbB4* with cardiovascular death [28] supported that these gene polymorphisms may have the potential to be prognostic biomarkers. Heart transplantation has been the most efficient treatment for end-stage HF. However, there is very limited finding regarding the biomarkers for survival from HF onset to heart transplantation. In one of the included studies, the findings showed that *COL1A1* may act as a biomarker for HF progression [23]. Plasma concentration of *COL1A1* was significantly negatively correlated with HF survival time. Moreover, a plasma *COL1A1* content threshold could be defined for the patients being transplanted within 1 year versus longer than 1 year. Three included studies showed that potential biomarkers were related to HF severity [37,55,68]. Akat et al. reported that circulating myomiRs may be useful for predicting the severity of HF [37]. The findings showed that myomiR changes in advanced HF reversed 3 and 6 months after LVAD implantation. The levels of the myomiRs mir-208a(1), mir-208b(1), mir-499(1), and mir-1-1(4) dropped as early as 3 months after the initiation of LVAD support, approaching normal levels. At LVAD explantation, the myomiR levels rose again with alterations comparable in magnitude to those observed at implantation. Two included studies reported that the plasma levels of miR-150-5p and miR-126 are associated with New York Heart Association (NYHA) class [55,68].

## 5. Limitations

This review explores the potential use of biomarkers in assisting in HF diagnosis and predicting prognosis. However, there are some limitations that need to be acknowledged: (1) Due to the heterogeneity of genetic components examined in the studies, pooled effects using a meta-analysis is not feasible. (2) Different ethnic groups involved in different studies may limit the generalizability of the findings. Therefore, further studies in different cohorts with different ethnicity are required. (3) Limited sample size especially of studies for the cardiac tissues may limit the detection of true effects. (4) The number of studies on biomarkers of HF is still limited when compared to traditional biomarkers, suggesting the need of more studies with a vigorous methodology to verify the findings and explore other biomarkers associated with HF.

## 6. Future Perspectives on Biomarker Research

As genome technologies evolve, individual genome sequencing can be made available to all patients using a simple saliva test. Understanding how genetic differences in individuals contribute to their susceptibility to cardiovascular diseases can help guide practitioners to give patients useful and pertinent advice for them to achieve a favorable outcome. Nowadays, large-scale genome-wide association studies and meta-analyses have provided powerful insights into polymorphisms that will be the key to efficiently diagnose diseases and adverse drug responses and direct the patients toward the drugs more likely to be of benefit based on their particular profile.

Genetic testing has been widely used to diagnose monogenic diseases. However, common diseases, such as type 2 diabetes and many neurodegenerative diseases, tend to be polygenic. Therefore, the concept of a polygenic risk score (PRS) is shown to have the potential for broad-scale clinical use. The study of specific biomarkers for specific heart diseases would feed into a growing body of evidence supporting a wider use of PRS. There are studies on the PRS and coronary artery disease (CAD), which is the most common form of heart disease and the most common cause of heart failure. Marston et al. and Damask et al. reported two studies of CAD PRS, both found evidence for the value of CAD PRS in predicting recurrent cardiovascular events; furthermore, they found that some subgroups might benefit from PCSK9 inhibitors. Another study using CAD patients from the UK Biobank, reported that using PRS could potentially help in preventing 7% more cardiovascular events when a targeted approach for screening (including PRS and traditional risk factors) is used. While these studies are very promising, there are limitations on the use of PRS in the management of patients with high PRS scores for CAD, and some have opined that further work is needed before PRS can be implemented clinically while others seem more optimistic and published a guide for the general cardiologist with the implication that PRS might soon be useful for clinical practice.

This review summarizes the first step of the use of biomarkers in HF diagnosis—the discovery of potential biomarkers. In the future, efforts are needed for qualification, verification, research assay optimization, biomarker validation, and commercialization because of the successful development of this area of research will represent a step forward in the individualization of diagnosis, therapy, and monitoring of HF.

## 7. Conclusions

Genetic variation may contribute to disease largely through the misregulation of genetic components. Examination of gene regulation thus becomes an important tool for disease diagnosis. Overall, this review demonstrated the potential use of transcriptome, miRNA, SNP, lncRNA, DNA methylation, and circRNA in HF with respect to diagnosis, prognosis, safety, specificity, and sensitivity. Given the complex physiology in HF, it is reasonable to expect that the future of biomarker testing lies in the application of multi-marker testing panels, precision medicine to improve HF care delivery, and the use of biomarkers in proteomics and metabolomics to further improve HF care.

## Figures and Tables

**Table 1:** Search strategy.

“Heart Failure”
AND
“transcriptome” OR “microRNA” OR “miRNA” OR “circular RNA” OR “epigenetic” OR “long noncoding RNA” OR “long non-coding RNA” OR “single nucleotide polymorphisms” OR “genome wide association studies” OR “GWAS” OR “polygenic risk score” OR “variants”
AND
“population” OR “cohort” OR “human” OR “patients” OR “cases” OR “subjects” OR “participants”
AND
“correlated” OR “correlation” OR “correlations” OR “associated” OR “association” OR “associations” OR “allele frequencies” OR “genotype frequencies” OR “differentially expressed” OR “upregulated” OR “downregulated”
AND
“genes” OR “gene” OR “biomarkers” OR “transcripts” OR “RNA”

**Table 2:** List of potential biomarkers of heart failure.

Biomarkers	Number of Samples	Ethnic Group	Risk of HF/Clinical Outcome (Sensitivity, Specificity)	Tissues Used	References
Gene (SNP)					
*HDC* (rs17740607)	1771	Chinese	CHF predisposition	Peripheral blood	[18]
*VDR*	235	Chinese	CHF predisposition	Peripheral blood	[19]
*ADA* (rs452159)	700	Chinese	CHF predisposition	Peripheral blood	[20]
*HRH3* (rs3787429)	687	Chinese	HF prognosis	Peripheral blood	[21]
*IL-17A* (rs8193037) *IL-17RA* (rs4819554)	>1000	Chinese	HF prognosis and mortality	Peripheral blood	[26]
*ErbB4* (rs1300941)	>1000	Chinese	HF prognosis and cardiovascular death	Peripheral blood	[28]
*MHRT* (rs7140721) (rs3729829) (rs3729825)	480	Chinese	HF predisposition and prognosis	Peripheral blood	[33]
*HSPB7* (rs1739843), *FRMD4B* (6787362)	4789	American	HF predisposition	Peripheral blood	[39]
*CLCNKA* (rs10927887)	5459	American	HF predisposition	Peripheral blood	[40]
*TGFBR3* (rs6996224)	3038	American	HFpEF predisposition	Peripheral blood	[42]
Chromosome 20p 12 (rs2207418)	2073	American	HF predisposition and mortality	Peripheral blood	[43]
Chromosome 15 (rs28714259)	2307	American	HF predisposition	Peripheral blood	[45]
*TNF-α*(−238)	193	Iranian	HF predisposition	Peripheral blood	[47]
*IL-4* (−590), *IL-4* (−33), *IL-4* (−1098)	182	Iranian	HF predisposition	Peripheral blood	[48]
*IL-1β* (−511)	183	Iranian	HF predisposition	Peripheral blood	[49]
*IL-2* (−330, +166)	195	Iranian	HF predisposition	Peripheral blood	[50]
IL-10 (−1082, −819, −592), *TGF-β1* (codon 10, codon 25)	197	Iranian	CHF predisposition	Peripheral blood	[51]
*KCNE1* (S38G)	933	Italian	HF predisposition	Peripheral blood	[52]
*AGTR1* (rs16860760) (rs389566) (rs5186)	352	Taiwanese	Diastolic HF prognosis	Peripheral blood	[62]
*MYBPC3* (rs2290149)	352	Taiwanese	Diastolic HF predisposition	Peripheral blood	[63]
*MIF* G173C (rs755622)	150	Egyptian	HF predisposition	Peripheral blood	[67]
*LEP* (Gln223Arg)	779	Czech	HF predisposition	Peripheral blood	[69]
Chr 1 (rs660240), chr 4 (rs17042102), chr 5 (rs11745324), chr 6 (rs4135240, rs55730499, rs140570886), chr 9 (rs1556516, rs600038), chr10 (rs4746140, rs17617337), chr 12 (rs4766578), chr16 (rs56094641)	977,323	Global	HF predisposition	Peripheral blood	[72]
*AGT* (M174, T235), ACE(D)	169	Canadian	HF predisposition	Peripheral blood	[74]
Transcriptomes					
*COL1A1*	169	Chinese	HF progression and survival time	Cardiac tissue and peripheral blood	[23]
*GAS5*, *TUG1*, and *Hotair*	20	Chinese	HF predisposition	Peripheral blood	[29]
*UNC93B1*	10	Chinese	HF prognosis	Epicardial adipose tissue	[36]
*STEAP4*, *SPARCL1*, *VSIG4*	27	Not mentioned	Prognosis of HF with right ventricular dysfunction	Right ventricular cardiac tissue	[41]
*WIPI1*	15	American	Discriminate HF with right ventricular dysfunction from healthy controls	Cardiac tissue	[46]
*SLC8A1*, *CHRNE*, *HCN2*, *BDKRB2*, *CACNA1G*	8	Italian	Discriminate HF with left ventricular dysfunction from healthy controls	Left ventricular cardiac tissue	[54]
*ANP*, *BNP*, *Slc8a1*, *CACNB2*, *MHC*	Not mentioned	German	HF prognosis	Cardiac tissue	[61]
*casp3*, *coll I*, *coll III*, and *TGF*	34	Chinese	HF predisposition	Peripheral blood	[71]
miRNA					
miR-3135b, miR-3908, miR-5571-5p	69	Chinese	HF predisposition and discriminate HFrEF and HFpEF	Peripheral blood	[14]
CDR1as, miR-135a, miR-135b, and HMOX-1	60	Chinese	HF predisposition	Peripheral blood	[15]
miRNA-21-5p, miRNA 30a-3p, miRNA 30a-5p, miRNA 155-5p, miRNA 216a, miRNA217	124	Chinese	HF predisposition	Peripheral blood	[16]
miR-195-3p	167	Chinese	HF predisposition	Peripheral blood	[22]
*COL1A1*	169	Chinese	HF progression and survival time	Cardiac tissue and peripheral blood	[23]
miR-660-3p, miR-665, miR-1285-3p, miR-4491	114	Chinese	HF predisposition and severity	Cardiac tissue and peripheral blood	[24]
hsa-miR-26b-5p, hsa-miR-8485, hsa-miR-940	20	Chinese	HF predisposition	Peripheral blood	[29]
miR-92b-5p	58	Chinese	Discriminate HFrEF from healthy controls	Peripheral blood	[30]
miR-30c	186	Chinese	HF predisposition	Peripheral blood	[31]
COX-2, miR-4649, miR-1297	147	Chinese	Discriminate nonischemic HF from healthy controls	Peripheral blood	[32]
mir-208a, mir-208b, mir499	171	American	Prognosis, severity of HF, prediction after LVAD implantation	Cardiac tissue and peripheral blood	[37]
miRNA-146a	60	USA	HF predisposition	Peripheral blood	[38]
miR129-5p	71	American	HF predisposition and severity	Peripheral blood	[44]
miR-150-5p	69	Italian	HF severity	Peripheral blood	[55]
let-7i-5p, miR-18a-5p, miR-18b-5p, miR-223-3p, miR-301a-3p, miR-423-5p miR-652-3p	198	Dutch	HF predisposition and prognosis	Peripheral blood	[56]
miR-423-5p	113	Dutch	HF predisposition	Peripheral blood	[57]
miR-16-5p, miR-106a-5p, miR-223-3p, miR-652-3p, miR-199a-3p, miR-18a-5p	124	Not mentioned	HF prognosis	Peripheral blood	[58]
miR-21, miR-129, miR212	Not mentioned	German	HF prognosis	Cardiac tissue	[61]
let-7b-5p, let-7c-5p, let-7e-5p, miR-122-5p, miR-21-5p, miR-16-5p, miR-17-5p, miR-27a-3p, miR-30a-5p, miR-30d-5p, miR-30e-5p, miR-130a-3p, miR-140-5p, miR-199a-5p, and miR-451a	17	Australian	HF predisposition	Aortic and coronary sinus blood	[65]
miR_103, miR_142_3p, miR_199a_3p, miR_23a, miR_27b, miR_324_5p, miR_342_3p miR_30b	287	New Zealanders	HF predisposition	Peripheral blood	[66]
miR126	60	Japanese	HF predisposition and severity	Peripheral blood	[68]
miR-1, miR-21, miR-23, miR-29, miR-130, miR-195, miR-199	34	Chinese	HF predisposition	Peripheral blood	[71]
miR-1233, miR-125a-5p, miR-183-3p, miR-190a, miR-193b-3p, miR-193b-5p, miR-211–5p, miR-494, miR-545–5p, miR-550a-5p, miR-638, miR-671-5p	176	Singaporeans	Distinguishing between HF and controls and between HFrEF and HFpEF	Peripheral blood	[73]
lncRNA					
*COL1A1*	169	Chinese	HF progression and survival time	Cardiac tissue and peripheral blood	[23]
*GAS5*, *TUG1* and *Hotair*	20	Chinese	HF predisposition	Peripheral blood	[29]
*CASC7*	186	Chinese	HF predisposition	Peripheral blood	[31]
*TUG1*	160	Chinese	HF predisposition and severity	Peripheral blood	[34]
ENST00000610659	10	Chinese	HF prognosis	Epicardial adipose tissue	[36]
*STEAP4*, *SPARCL1*, *VSIG4*	27	Not mentioned	Prognosis of HF with right ventricular dysfunction	Right ventricular cardiac tissue	[41]
*CDKN2B-AS1*, *HOTAIR*, LOC285194	46	Italian	HF progression	Left ventricular cardiac tissue	[53]
*SLC8A1*, *CHRNE*, *HCN2*, *BDKRB2*, *CACNA1G*	8	Italian	Discriminate HF with left ventricular dysfunction from healthy controls	Left ventricular cardiac tissue	[54]
*Heat2*	139	German	Discriminate HFrEF from healthy controls	Cardiac tissue and peripheral blood	[59]
DNA methylation					
*SLC2A1*, *MPV17L*, *PLEC*	47	Chinese	HF predisposition	Peripheral blood	[25]
*METTL3*, *METTL4*, *KIAA1429*, *FTO*, *YTHDF2*	40	Chinese	HF predisposition	Peripheral blood	[35]
*B9D1*	72	German	HF predisposition	Cardiac tissue and peripheral blood	[60]
*HDAC9*	20	Australian	HF predisposition	Peripheral blood	[64]
*HEY2*, *MSR1*, *MYOM3*, *COX17*, *MMP2*, *CTGF*, miR-24-1, miR-155	78	Irish	HF predisposition	Peripheral blood	[70]
circRNA					
hsa_circ_0099476, hsa_circ_0001312, hsa_circ_0005158, hsa_circ_0029696, hsa_circ_0040414	89	Chinese	HF predisposition	Peripheral blood	[17]
has_circ_0112085, hsa_circ_0062960, hsa_circ_0053919, hsa_circ_0014010	60	Chinese	HF predisposition	Peripheral blood	[27]

## Supplementary Materials

The following are available online at https://www.mdpi.com/article/10.3390/ijerph18115904/s1, Table S1: The ratings of the reporting quality of the included studies based on the STROBE checklist, Table S2: The characteristics of the included studies.
