# Supplementary material for: Exploration of Potential Genetic Biomarkers for Heart Failure: A Systematic Review
Source: Int J Environ Res Public Health. 2021 May 31;18(11):5904. doi: 10.3390/ijerph18115904 (PMC8198957; doi:10.3390/ijerph18115904)
Supplement: Supplementary file 1 [file ijerph-18-05904-s001.zip › Supplementary Table S1_IJERPH 1148215.pdf]

Supplementary Table S1. The ratings of the reporting quality of the included studies based on the STROBE checklist

Item	Akat et al., 2014 [37]	Bain et al., 2020 [64]	Beg et al., 2017 [38]	Bienertová-Vaskǔ et al., 2009 [69]	Boeckel et al., 2019 [59]	Cappola et al., 2010 [39]	Cappola et al., 2011 [40]	Chen et al., 2018 [14]	Chen et al., 2020 [15]
**Study design described**	Yes	No	No	Yes	No	Yes	No	No	Yes
**Study setting described**	Yes	Yes	Yes	Yes	No	Yes	Yes	Yes	Yes
**Eligibility and criteria of participants described**	No	Yes	Yes	Yes	No	Yes	Yes	Yes	Yes
**Outcomes and variables defined**	Yes	Yes	Yes	Yes	Yes	Yes	Yes	Yes	Yes
**Methods of measurements described**	Yes	Yes	Yes	Yes	Yes	Yes	Yes	Yes	Yes
**Attempt to address potential bias**	No	No	No	No	No	Yes	No	No	No
**Rationale of study size described**	No	No	No	No	No	No	No	No	No
**Statistical methods described**	Yes	No	Yes	Yes	Yes	Yes	Yes	Yes	Yes
**Number of participants at each stage of study reported**	Yes	Yes	Yes	Yes	Yes	Yes	Yes	Yes	Yes
**Characteristics of participants reported**	No	Yes	Yes	Yes	Yes	Yes	No	Yes	No
**Outcome data reported**	Yes	Yes	Yes	Yes	Yes	Yes	Yes	Yes	Yes
**Adjusted and/or unadjusted estimates reported**	Yes	Yes	Yes	Yes	Yes	Yes	Yes	Yes	Yes
**Score (Max. 12)**	**8**	**8**	**9**	**10**	**7**	**11**	**8**	**9**	**9**

Table S1 Cont.

Item	di Salvo et al., 2015 [41]	Ding et al., 2020 [16]	EI-Mahdy et al., 2021 [67]	Ellis et al., 2013 [66]	Fatini et al., 2010 [52]	Fukushima et al., 2010 [68]	Glezeva et al., 2019 [70]	Greco et al., 2016 [53]	Han et al., 2020 [17]
**Study design described**	Yes	No	Yes	Yes	No	No	No	Yes	Yes
**Study setting described**	Yes	Yes	Yes	Yes	Yes	No	Yes	Yes	Yes
**Eligibility and criteria of participants described**	Yes	No	Yes	Yes	Yes	Yes	No	Yes	Yes
**Outcomes and variables defined**	Yes	Yes	Yes	Yes	Yes	Yes	Yes	Yes	Yes
**Methods of measurements described**	Yes	Yes	Yes	Yes	Yes	Yes	No	Yes	Yes
**Attempt to address potential bias**	Yes	No	No	Yes	Yes	Yes	No	No	No
**Rationale of study size described**	No	No	Yes	No	No	No	No	No	No
**Statistical methods described**	Yes	Yes	Yes	Yes	Yes	Yes	No	Yes	Yes
**Number of participants at each stage of study reported**	Yes	Yes	Yes	Yes	Yes	Yes	Yes	Yes	Yes
**Characteristics of participants reported**	Yes	Yes	Yes	Yes	Yes	Yes	Yes	Yes	No
**Outcome data reported**	Yes	Yes	Yes	Yes	Yes	Yes	Yes	Yes	Yes
**Adjusted and/or unadjusted estimates reported**	Yes	Yes	Yes	Yes	Yes	Yes	Yes	Yes	Yes
**Score (Max. 12)**	**10**	**9**	**10**	**10**	**10**	**9**	**6**	**10**	**9**

Table S1 Cont.

Item	Hao et al., 2019 [19]	He et al., 2014 [20]	He et al., 2015 [18]	He et al., 2016 [21]	He et al., 2017 [22]	Hedayat et al., 2018 [47]	Hua et al., 2020 [23]	Kao et al., 2017 [42]	Lai et al., 2015 [71]
**Study design described**	Yes	No	Yes	Yes	Yes	Yes	Yes	No	No
**Study setting described**	Yes	Yes	Yes	Yes	Yes	Yes	Yes	Yes	Yes
**Eligibility and criteria of participants described**	Yes	Yes	Yes	Yes	Yes	Yes	Yes	Yes	No
**Outcomes and variables defined**	Yes	Yes	Yes	Yes	Yes	Yes	Yes	Yes	Yes
**Methods of measurements described**	Yes	Yes	Yes	Yes	Yes	Yes	Yes	Yes	Yes
**Attempt to address potential bias**	No	No	No	No	Yes	Yes	No	No	No
**Rationale of study size described**	No	No	No	No	No	No	No	No	No
**Statistical methods described**	Yes	Yes	Yes	Yes	Yes	Yes	Yes	Yes	Yes
**Number of participants at each stage of study reported**	Yes	Yes	Yes	Yes	Yes	Yes	Yes	Yes	Yes
**Characteristics of participants reported**	No	Yes	Yes	Yes	Yes	No	Yes	Yes	Yes
**Outcome data reported**	Yes	Yes	Yes	Yes	Yes	Yes	Yes	Yes	Yes
**Adjusted and/or unadjusted estimates reported**	Yes	Yes	Yes	Yes	Yes	Yes	Yes	Yes	Yes
**Score (Max. 12)**	**9**	**9**	**10**	**10**	**11**	**10**	**10**	**9**	**9**

Table S1 Cont.

Item	Li et al., 2015 [24]	Li et al., 2017 [25]	Mahmoudi et al., 2014 [48]	Mahmoudi et al., 2016 [49]	Mahmoudi et al., 2018 [50]	Mahmoudi et al., 2019 [51]	Marques et al., 2016 [65]	Meder et al., 2017 [60]	Ovchinnikova et al., 2016 [56]
**Study design described**	No	Yes	No	No	Yes	Yes	Yes	Yes	Yes
**Study setting described**	Yes	Yes	Yes	Yes	Yes	Yes	Yes	Yes	Yes
**Eligibility and criteria of participants described**	Yes	No	Yes	Yes	Yes	Yes	Yes	Yes	Yes
**Outcomes and variables defined**	Yes	Yes	Yes	Yes	Yes	Yes	Yes	Yes	Yes
**Methods of measurements described**	Yes	Yes	Yes	Yes	Yes	Yes	Yes	Yes	Yes
**Attempt to address potential bias**	No	No	No	No	Yes	No	No	No	No
**Rationale of study size described**	No	No	No	No	No	No	No	No	No
**Statistical methods described**	Yes	Yes	Yes	Yes	Yes	Yes	Yes	Yes	Yes
**Number of participants at each stage of study reported**	Yes	Yes	Yes	Yes	Yes	Yes	Yes	Yes	Yes
**Characteristics of participants reported**	Yes	No	No	No	Yes	Yes	Yes	No	Yes
**Outcome data reported**	Yes	Yes	Yes	Yes	Yes	Yes	Yes	Yes	Yes
**Adjusted and/or unadjusted estimates reported**	Yes	Yes	Yes	Yes	Yes	Yes	Yes	Yes	Yes
**Score (Max. 12)**	**9**	**8**	**8**	**8**	**11**	**10**	**10**	**9**	**10**

Table S1 Cont.

Item	Parsa et al., 2010 [43]	Ramachandran et al., 2017 [44]	Sandip et al., 2016 [26]	Schiano et al., 2017 [54]	Schneider et al., 2017 [45]	Scrutinio et al., 2017 [55]	Shah et al., 2020 [72]	Sun et al., 2020 [27]	Thum et al., 2007 [61]
**Study design described**	No	Yes	Yes	Yes	Yes	Yes	Yes	No	Yes
**Study setting described**	Yes	Yes	Yes	Yes	Yes	Yes	Yes	Yes	Yes
**Eligibility and criteria of participants described**	Yes	No	Yes	No	Yes	Yes	No	No	No
**Outcomes and variables defined**	Yes	Yes	Yes	Yes	Yes	Yes	Yes	Yes	Yes
**Methods of measurements described**	Yes	Yes	Yes	Yes	Yes	Yes	Yes	Yes	Yes
**Attempt to address potential bias**	No	No	No	No	No	No	No	No	Yes
**Rationale of study size described**	No	No	No	No	No	No	No	No	No
**Statistical methods described**	No	Yes	Yes	Yes	Yes	Yes	Yes	Yes	Yes
**Number of participants at each stage of study reported**	Yes	Yes	Yes	Yes	Yes	Yes	Yes	Yes	Yes
**Characteristics of participants reported**	Yes	Yes	Yes	Yes	No	Yes	No	Yes	No
**Outcome data reported**	Yes	Yes	Yes	Yes	Yes	Yes	Yes	Yes	Yes
**Adjusted and/or unadjusted estimates reported**	Yes	Yes	Yes	Yes	Yes	Yes	Yes	Yes	Yes
**Score (Max. 12)**	**8**	**9**	**10**	**9**	**9**	**10**	**9**	**9**	**9**

Table S1 Cont.

Item	Tijsen et al., 2010 [57]	Tzimas et al., 2019 [46]	Vegter et al., 2016 [58]	Wang et al., 2016 [28]	Wang et al., 2019 [29]	Wong et al., 2015 [73]	Wu et al., 2009 [62]	Wu et al., 2012 [63]	Wu et al., 2018 [30]
**Study design described**	No	Yes	Yes	No	Yes	Yes	Yes	Yes	No
**Study setting described**	Yes	Yes	Yes	Yes	Yes	Yes	Yes	Yes	Yes
**Eligibility and criteria of participants described**	Yes	No	Yes	Yes	Yes	Yes	Yes	Yes	Yes
**Outcomes and variables defined**	Yes	Yes	Yes	Yes	Yes	Yes	Yes	Yes	Yes
**Methods of measurements described**	Yes	Yes	Yes	Yes	Yes	Yes	Yes	Yes	Yes
**Attempt to address potential bias**	No	Yes	No	Yes	Yes	No	No	No	No
**Rationale of study size described**	No	No	No	No	No	No	No	No	No
**Statistical methods described**	Yes	Yes	Yes	Yes	Yes	Yes	Yes	Yes	Yes
**Number of participants at each stage of study reported**	Yes	Yes	Yes	Yes	Yes	Yes	Yes	Yes	Yes
**Characteristics of participants reported**	Yes	Yes	Yes	Yes	Yes	Yes	Yes	Yes	Yes
**Outcome data reported**	Yes	Yes	Yes	Yes	Yes	Yes	Yes	Yes	Yes
**Adjusted and/or unadjusted estimates reported**	Yes	Yes	Yes	Yes	Yes	Yes	Yes	Yes	Yes
**Score (Max. 12)**	**9**	**10**	**10**	**10**	**11**	**10**	**10**	**10**	**9**

Table S1 Cont.

Item	Xu et al., 2020 [31]	Yan et al., 2020 [32]	Zakrzewski-Jakubiak et al., 2008 [74]	Zhang et al., 2020 [33]	Zhang et al., 2021 [34]	Zhang et al., 2021a [35]	Zheng et al., 2019 [36]
**Study design described**	Yes	Yes	Yes	Yes	No	Yes	No
**Study setting described**	Yes	Yes	Yes	Yes	Yes	Yes	Yes
**Eligibility and criteria of participants described**	No	Yes	Yes	Yes	Yes	Yes	Yes
**Outcomes and variables defined**	Yes	Yes	Yes	Yes	Yes	Yes	Yes
**Methods of measurements described**	Yes	Yes	Yes	Yes	Yes	Yes	Yes
**Attempt to address potential bias**	No	No	No	No	No	No	No
**Rationale of study size described**	No	No	No	No	No	No	No
**Statistical methods described**	Yes	Yes	Yes	Yes	Yes	No	Yes
**Number of participants at each stage of study reported**	Yes	Yes	Yes	Yes	Yes	Yes	Yes
**Characteristics of participants reported**	Yes	Yes	Yes	Yes	Yes	No	Yes
**Outcome data reported**	Yes	Yes	Yes	Yes	Yes	Yes	Yes
**Adjusted and/or unadjusted estimates reported**	Yes	Yes	Yes	Yes	Yes	Yes	Yes
**Score (Max. 12)**	**9**	**10**	**10**	**10**	**9**	**8**	**9**
