# Supplementary material for: Exploration of Potential Genetic Biomarkers for Heart Failure: A Systematic Review
Source: Int J Environ Res Public Health. 2021 May 31;18(11):5904. doi: 10.3390/ijerph18115904 (PMC8198957; doi:10.3390/ijerph18115904)
Supplement: Supplementary file 1 [file ijerph-18-05904-s001.zip › Supplementary Table S2_IJERPH 1148215.pdf]

Supplementary Table S2. The characteristics of the included studies

Author/Year/Country	Sample type/sample size	Genetic components assessed	Major findings
Akat et al., 2014 [37]; USA	• Myocardial tissue;<br>- advanced HF (n=34)<br>- healthy control (n=8)<br>- fetuses (n=5)<br><br>• Plasma;<br>- advanced HF (n=24)<br>- stable HF (n=14)<br>- healthy control (n=13)<br><br>• Serum;<br>- advanced HF (n=14)<br>- healthy control (n=4)	miRNA	• In advanced HF patients, cardiac-specific (mir-208b, mir-208a, mir-499) and muscle-specific (mir-1-1, mir-133b) circulating miRNAs (myomirs) increased up to 140-fold when compared with healthy controls, which coincided with a similar increasing trend in cardiac troponin I (cTnl) protein<br><br>• In stable HF patients, circulating miRNA of myomir showed less than 5-fold difference compared with healthy controls<br><br>• The levels of myomirs miRNA mir-208b, mir-208a, mir-499 and mir-1-1 in advanced HF reversed 3 and 6 months after LVAD implantation (p<0.05)
Bain et al., 2020 [64]; Australia	• Peripheral blood;<br>- CHF patients (n=10)<br>- Diabetic controls (n=10)	DNA methylation	• DMRs with reduced methylation in HF, 19 were protein-coding genes (ABCB1, ARID5B,CAV2, DIS3, DOPEY2, GREM1, HDAC9, HS6ST3, IQCA1,JARID2, MYO1H, NFIA, OPA1, PACRG, RPS6KA5, RUNDC3B,SOS2, SOX6, and TC2N) and four non-coding RNA genes<br>• DMRs with increased methylation in HF, 19protein-coding genes were identified (ACSS2, DAAM2,GPA33/DUSP27, EDEM1, FAM212B, FRMD4A, KCNK10,MICALCL, MYO5A, PARP4, PDSS2, PLA2G4E, RALGAPA1,RGS9, SCAMP1, and

			TRPM6) and 12 non-coding RNA genes<br>• HDAC9DMRportion analysed, within intron 19 (chr7:18 834 681–18 834 888), had high methylation in the control group (91.8%), and the HF group had lower methylation at two of the four CpG sites analysed. Sites c34 and c49 were lower in HF by 0.62% and 0.99%, respectively
Beg et al., 2017 [38]; USA	• Peripheral blood;<br>- HF (n=40)<br>- Control (n=20)	miRNA	• Average fold-change for plasma levels of miR-486 and miR-146a showed a trend towards elevated expression in patients with heart failure (1.1 ± 0.27 and 2.3 ± 0.79 respectively) but was not statistically significant (0.82 and 0.14 respectively)<br>• Circulating exosomes of heart failure patients contained a significantly elevated levels of miR-146a compared to controls (2.46 ± 0.51, p = 0.05) and also for miR-486 (3.0 ± 0.95, p = 0.14)
Bienertová-Vasků et al., 2009 [69]; Czech Republic	• Peripheral blood;<br>- HF (n=372)<br>- Control (n=407)	SNP	• R allele of LEP Gln223Arg are more frequent in CHF below 56 years of age (P = 0.0002, odds ratio [OR] = 1.29, 95% confidence interval [CI] = 1.089–1.549)<br>• LEP Gln223Arg polymorphism is associated with higher risk for NYHA class IV (P = 0.0000001, OR = 2.10, 95% CI = 1.56–2.84) and for LVEF (P = 0.002, OR = 4.05, 95% CI = 1.36–10.06)<br>• LEPR Gln223Arg showed an independent prediction role for NYHA IV in IHD patients (P = 0.0001, OR = 2.50, 95% CI = 1.69–3.82) and for NYHA IV (P = 0.007,

			OR = 2.04, 95% CI = 1.20–3.84) and left ventricular ejection fraction (LVEF) (P = 0.004, OR = 11.87, 95% CI = 2.08–55.6)
Boeckel et al., 2019 [59]; Germany	• PBMC;<br>- Cohort 1: HF (n=4), healthy control (n=4)<br>- Cohort 2: HF (n=16), healthy control (n=8)<br>- Cohort 3: HF (n=69), healthy control (n=38)	lncRNA	• 110 potential lncRNAs were revealed in cohort 1 and up-regulated lncRNA Heat2 was selected for further characterized<br>• Elevation of Heat2 in HF patients were validated in cohort 2 and cohort 3<br>• Heat2 expression level were significantly higher in basophils (24-fold) and eosinophils (2184-fold) of HF patients<br>• Invasion and transmigration capacities of PBMCs were significantly increased after elevation of Heat2 expression (p<0.05)<br>• Cell adhesion of PBMCs to endothelial cells significantly reduced after siRNA-mediated knockdown of Heat2 (p<0.05)

Cappola et al., 2010 [39]; USA	• Peripheral blood;<br>*Stage 1:*<br>- HF (n=1590)<br>- Control (n=577)<br>*Stage 2:*<br>- HF (n=308)<br>- Control (n=2314)	SNP	• Two SNPs surpassed the threshold for significance association with HF in primary analyses (rs1738943[HSPB7], P =2.8×10−5 and rs6787362[FRMD4B], P=4.5×10−5)<br>• rs1738943(HSPB7) and rs6787362(FRMD4B) showed significant associations with HF with P-values 3.09×10−6 for rs1739843 and 6.09×10−6 for rs6787362<br>• In subgroup analyses, rs1739843 associated with both ischemic and non-ischemic heart failure, whereas rs6787362 associated principally with ischemic heart failure

Cappola et al., 2011 [40]; USA	• Peripheral blood; *Primary cohort:* - HF (n=1113) - Control (n=603) *Secondary cohort:* - HF (n=755) - Control (n=1887) *Tertiary cohort:* - HF (n=785) - Control (n=316)	SNP	• Nine SNPs of CLCNKA had significant associations with heart failure ($P < 0.001$, corresponding to a Bonferroni-adjusted $P < 0.05$) • rs10927887 was overrepresented in heart failure (≈0.56 vs. ≈0.48 in controls; $P = 4.4 \times 10{-}4$; secondary cohort). Combined analysis from both study cohorts gives a P value for association of $2.25 \times 10{-}6$ • In age- and gender-adjusted models, the CLCNKA Gly83 allele was associated with all-cause heart failure in all three cohorts, with an overall 1.26-fold increase in risk per allele copy ($P = 3.8 \times 10{-}6$)

Chen et al., 2018 [14]; China	• Peripheral blood; *Screening cohort:* - HF (n=13) - Healthy control (n=3) *Validation cohort:* - HF (n=33) - Healthy control (n=20)	miRNA	• In the HF group, three miRNAs' expressions were increased and nine miRNAs were decreased compared with the control group • In validation cohort, results demonstrated a 3.3-fold increase in miR-3135b expression (P<0.001), a 1.4-fold increase in miR-3908 expression (P<0.001), and a 2.0-fold increase in miR-5571–5p expression (P<0.001) in HF patients • The ROC analysis showed that miR-3135b, miR-3908, and miR-5571-5p had significant AUC (0.86-1.00) • NT-proBNP concentration (P = 0.30) in HFrEF patients was similar to that in HFpEF patients. The expressions of miR-3135b (P < 0.05) and miR-3908 (P < 0.05) were found to differ significantly between HFrEF and HFpEF patients.

Chen et al., 2020 [15]; China	• Peripheral blood;<br>- CHF patients (n=30)<br>- Healthy controls (n=30)	Circ RNA, miRNA, transcriptome	• CDR1as, the circular RNA:<br>- The plasma level of CDR1as (a circular RNA that acts as a miRNA inhibitor) was found to be significantly higher among chronic HF patients compared to controls ($p<0.05$).<br>- Subjects with higher plasma level of CDR1as were at higher NYHA class of their heart failure.<br>- ROC curve analysis revealed that the area under the ROC curve for CDR1as is 0.84 ($p<0.001$), indicating the potential of plasma CDR1as level as diagnostic marker for HF.<br>• miR-135a and miR-135b, the miRNAs:<br>- Expression level of both miRNAs were decreased in CHF patients compared to controls ($p\leq0.01$).<br>- Subjects with higher plasma level of CDR1as were at lower NYHA class of their heart failure.<br>- ROC curve analysis revealed that the area under the ROC curve for miR-135a and miR-135b were 0.82 (95% CI: 0.71-0.93) and 0.66 (95% CI: 0.52-0.80) respectively (both $p<0.01$), also indicating the potential of the plasma level of both miRNAs to be used as diagnostic markers for HF.<br>• HMOX-1:<br>- Expression level of Heme oxygenase-1 (HMOX-1) gene were increased in chronic HF patients compared to controls ($p<0.05$)<br>- Subjects with higher plasma level of HMOX-1 were at higher NYHA class of their heart failure.

			- ROC curve analysis revealed that the area under the ROC curve for CDR1as is 0.85 (95% CI: 0.76-0.95; p<0.01), indicating the potential of plasma CDR1as level as diagnostic marker for HF<br>• Correlations between plasma levels of CDR1as, miR-135a, miR-135b and HMOX-1:<br>- Plasma level of CDR1as negatively correlated with that of miR-135a (R=-0.72, p<0.01) and miR-135b (R=-0.88, p<0.01)<br>- Plasma level of CDR1as positively correlated with that of HMOX-1 (R=0.84, p<0.01)<br>- Plasma level of HMOX-1 negatively correlated with that of miR-135a (R=-0.57, p<0.01) and miR-135b (R=-0.67, p<0.01)

di Salvo et al., 2015 [41]; USA	• Right ventricular myocardial tissue:<br>- ischemic HF (n=11)<br>- non-ischemic HF (n=11)<br>- echocardiographic dysfunction (n=11)<br>- echocardiographic normal function (n=11)<br>- unused donors (n=5)	Transcriptome, lncRNA	• DEGs found in different comparisons:<br>- 993 DEGs (unused donor RV vs nonischemic HF RV)<br>- 831 DEGs (unused donor RV vs ischemic HF RV)<br>- 848 DEGs (unused donor RV vs echocardiographic dysfunction)<br>- 904 DEGs (unused donor RV vs echocardiographic normal function)<br>• 10 transcripts were significantly reduced in HF RV group:<br>- SERPINA3, SERPINA5, LCN6, LCN10, STEAP4, AKR1C1, STAC2, SPARCL1, VSIG4, F8<br>• STEAP4, SPARCL1 and VSIG4 were significantly down-regulated in RV versus LV end-stage human myocardium ($p<0.05$)

Ding et al., 2020 [16]; China	• Peripheral blood;<br>- HF patients (n=62)<br>- Healthy controls (n=62)	miRNA	• In the heart failure samples, the expression of miRNA-21-5p, miRNA 30a-3p, miRNA 30a-5p, miRNA 155-5p, miRNA 216a and miRNA217 have a significant upward trend (p<0.05)<br>• ROC curve evaluation statistics show that AUC values of six miRNA is 0.660-0.944)

El-Mahdy et al., 2021 [67]; Egypt	• Peripheral blood;<br>- HF patients (n=90)<br>- Healthy controls (n=60)	SNP	• A SNP in the macrophage migration inhibitory factor (MIF) gene (rs755622) is likely to be related to HF risk.<br>• Compared to subjects harbouring the CC genotype variant only, those with the GG variant in the rs755622 SNP of the MIF gene would be at greater risk of HF (OR: 4.25; 95% CI: 0.98-18.45; p=0.047).<br>• The G allele in the rs755622 SNP of the MIF gene increases the risk of HF. although the difference was not significant (OR: 3.22; 95% CI: 0.77-13.42; p=0.092)<br>• However, the C allele in this SNP decreases HF risk (OR: 0.51; 95% CI: 0.26-0.99; p=0.046)<br>• Multivariate analysis showed that the GG genotype of MIF gene was an independent risk factor for HF (OR: 4.6; 95% CI: 1.1-20.4; p<0.05)
Ellis et al., 2013 [66]; New Zealand	• Peripheral blood;<br>*Screening study:*<br>- HFrEF (n=16)<br>- HFpEF (n=16)<br>- HF (n=32)<br>- COPD (n=15)<br>- Control (n=14)<br>*Validation:*<br>- HFrEF (n=22)<br>- HFpEF (n=22)<br>- HF (n=44)<br>- COPD (n=32)<br>- Other breathless (n=59)<br>- Control (n=15)	miRNA	• MiR-103 [area under the curve (AUC) = 0.642, P = 0.007], miR-142-3p (AUC = 0.668, P = 0.002), miR-199a-3p (AUC = 0.668, P = 0.002), miR-23a (AUC = 0.637, P = 0.010), miR-27b (AUC = 0.642, P = 0.008), miR-324-5p (AUC = 0.621, P = 0.023), and miR-342-3p (AUC = 0.644, P = 0.007) were associated with HF diagnosis in regression and receiver operating characteristic (ROC) analyses<br>• miR-103, miR-142-3p, miR-30b, and miR-342-3p, were differentially expressed between HF and controls, chronic obstructive pulmonary disease (COPD), and other breathless patients (P = 0.002–0.030)

Fatini et al., 2010 [52]; Italy	• Peripheral blood;;<br>*Cohort in Florence:*<br>- HF (n=195)<br>- healthy control (n=352)<br>*Cohort in Pisa:*<br>- HF (n=186)<br>- healthy control (n=200)	SNP	• Significant difference in genotype distribution (p=0.002) and allele frequency (p=0.0008) between HF and controls was observed for KCNE1 S38G SNP in Florence cohort<br>• Significant difference in genotype distribution (p=0.01) and allele frequency (p=0.004) between HF and controls was observed for KCNE1 S38G SNP in Pisa cohort<br>• No difference in genotype distribution (p=0.4) and allele frequency (p=0.3) for the *KCNE1* S38G SNP according to functional New York Heart Association class was found<br>• No significant difference was observed between KCNE1 S38G SNP and the parameters related to the severity of HF, such as ejection fraction (EF) (p=0.62), left ventricular end-diastolic diameter (LVEDD) (p=0.64) and left ventricular end-systolic diameter (LVESD) (p=0.63)
Fukushima et al., 2011 [68]; Japan	• Peripheral blood;<br>- HF patients (n=43)<br>- Healthy controls (n=17)	miRNA	• Plasma concentrations of miR-126 were highly and negatively correlated with age (r2=0.52; P=0.0006; N=17) in the control group<br>• Plasma concentrations of miR126 were significantly different among the control and NYHA II-IV groups<br>• Plasma concentrations of miR-126 were negatively correlated with BNP (r2=0.25; P=0.0003; N=43) in the NYHA II-IV groups<br>• plasma concentrations of miR-126 were up-regulated with improvement of the NYHA class from IV to III

Glezeva et al., 2019 [70]; Ireland	• Peripheral blood;<br>- HF patients (n=39)<br>- Healthy controls (n=39)	DNA methylation	• In hypertrophic obstructive cardiomyopathy (HOCM) group:<br>- 4 protein-coding genes (3 hypermethylated and 1 hypomethylated)<br>- 1 ncRNA (hypomethylated)<br>• In DCM group:<br>- 131 protein-coding genes (13 hypermethylated and 118 hypomethylated)<br>- 17 ncRNA (3 hypermethylated and 14 hypomethylated)<br>• In ISCM group:<br>- 51 protein-coding genes (8 hypermethylated and 43 hypomethylated)<br>- 5 ncRNA (3 hypermethylated and 2 hypomethylated)<br>• HEY2 and MSR1 were significantly hypermethylated in HOCM (15.81%, P=0.006 and 19.87%, P=0.044) with gene expression significantly reduced by 0.53-fold (P=0.001) and 0.42-fold (P=0.003), respectively, in HOCM group<br>• MYOM3 and COX17 were hypermethylated in ischemic cardiomyopathy (ISCM) (21.25%, P=0.003 and 25.99%, P=0.046), and their transcript levels were significantly reduced by 0.74-fold (P=0.019) and 0.49-fold (P=0.001), respectively.<br>• MMP2 was significantly hypomethylated in DCM (14.45%, P=0.032). Expression levels of MMP2 were increased by 2.67-fold in DCM (P=0.003)<br>• CTGF—in ISCM (17.52%, P=0.00003) and DCM (11.42%, P=0.019) at 2 neighboring DMRs.

			CTGF was upregulated by 2.85-fold in ISCM (P=0.005) and 3.33-fold in DCM (P=0.011)<br>• The expression of miR-24-1 was significantly reduced by 0.81-fold in the ISCM group only (P=0.031)<br>• MiR-155 expression was increased by 1.63-fold in ISCM patients (P=0.030)
Greco et al., 2016 [53]; Italy	• Myocardial tissue;<br>- HF (n=13)<br>- control (n=12)<br><br>• PBMCs;<br>- HF (n=25)<br>- control (n=18)	lncRNA	• 10 lncRNAs were up-regulated and 3 lncRNAs were down-regulated in HF patients compared with control subjects (p<0.05)<br>• 9 lncRNAs (CDKN2B-AS1, EGOT, H19, HOTAIR, LOC285194, RMRP, RNY5, SOX2-OT and SRA1) were validated to be significantly modulated in more severe end-stage HF patients<br>• 3 lncRNAs (CDKN2B-AS1/ANRIL, HOTAIR and LOC285194/TUSC7) showed similar modulation in PBMCs and heart tissue
Han et al., 2020 [17]; China	• Peripheral blood;<br>*CircRNA profiling analysis:*<br>- HF (n=45)<br>- healthy control (n=44)<br>*Validation by real-time PCR:*<br>- HF (n=40)<br>- healthy control (n=40)	CircRNA	• Compared with the control group, 29 circRNAs in the HF group were significantly up-regulated and 27 were significantly down-regulated<br>• The levels of hsa_circ_0097435 (p < 0.01), hsa_circ_0099476 (p < 0.01), hsa_circ_0001312 (p < 0.01), hsa_circ_0005158 (p < 0.01), hsa_circ_0029696 (p < 0.01), and hsa_circ_0040414 (p < 0.01) were significantly higher in the HF group than in the control group<br>• Significantly higher levels of hsa_circ_0097435 (p < 0.05) were detected in exosomes from patients with HF than in those from normal volunteers

He et al., 2015 [18]; China	• Peripheral blood;<br>*Xián cohorts:*<br>- CHF (n=439)<br>- control (n=467)<br>*Kunming cohort:*<br>- CHF (n=413)<br>- control (n=452)	SNP	• There is a significant difference in both genotype and allele distributions between patients with CHF and controls in Xi'an population for rs17740607 at Bonferroni-corrected p level<br>• A allele was more frequent in control group than in patients with CHF and exhibited protective effect against CHF<br>• rs17740607 polymorphism was significantly and independently associated with the predisposition to CHF under a dominant and additive but not recessive model in both populations<br>• a significantly greater prevalence of the rs17740607G variant was observed in CHF hypertensive patients, diabetic patients, and smokers than in controls in both populations<br>• the plasma histamine levels in rs17740607 variant genotype CHF or control groups were significantly lower than those in the corresponding wild-type groups in both populations
Hao et al., 2019 [19]; China	• Peripheral blood;<br>- CHF (n=145)<br>- control (n=90)	SNP	• CHF patients displayed significantly lower levels of plasma vitamin D3 compared to healthy controls ($P < 0.0001$)<br>• 25-OH vitamin D3 deficient were more prevalent in chronic heart failure patients (28%) compared to healthy controls (15%) ($P = 0.003$)<br>• Prevalence of heterozygous mutant (Ff) and minor allele (f) for FokI polymorphism was significantly higher in chronic heart failure patients compared to healthy controls

			(Ff: P < 0.0001, OR = 3.42; f: P < 0.0001, OR = 2.45)<br>• For TaqI gene polymorphism, Tt genotype and allele “t” were more frequent in patient then controls (Tt: P = 0.002, OR = 2.35; t: P = 0.007, OR = 1.86)<br>• In combination analysis, subjects with Ff/sufficient, Ff/insufficient, ff/insufficient, and Ff/deficient were significantly higher in chronic heart failure patients compared to healthy controls. In contrast, the prevalence of FF/insufficient combination was higher in controls compared to patients. Tt/sufficient, Tt/insufficient, tt/insufficient, TT/deficient, and Tt/deficient were significantly higher in CHF patients when compared to healthy controls. However, TT/insufficient combination was more frequent in controls than patients
He et al., 2014 [20]; China	• Peripheral blood;<br>- HF (n=300)<br>- healthy control (n=400)	SNP	• rs452159 polymorphism in ADA gene was significantly associated with susceptibility to CHF under the dominant model (p = 0.013, OR = 1.537, 95% CI = 1.10–2.16), after adjustment for age, sex, and traditional cardiovascular risk factors<br>• The genotype distribution and allele frequency of the rs452159 did not differ significantly with functional NYHA class<br>• No association was found between parameters related to HF severity and genotype for the rs452159 variant

He et al., 2016 [21]; China	• Peripheral blood;<br>- HF (n=333)<br>- healthy control (n=354)	SNP	• rs3787429 polymorphism of HRH3 gene was significantly correlated to HF risk on both genotype and allele (p<0.001, resp.)<br>• T allele of rs3787429 was more frequent in control group than in HF patients (adjusted OR, 0.608; 95% CI, 0.470-0.786; p<0.001)<br>• After adjustment, rs3787429 polymorphism was significantly and independently associated with HF under a dominant (OR, 0.455; 95% CI, 0.322-0.642; p<0.001) and additive model (OR, 0.662; 95% CI, 0.523-0.838; p=0.001), but not recessive model (OR, 0.835; 95% CI, 0.523-1.333; p=0.450)
He et al., 2017 [22]; China	• Peripheral blood;<br>*miR sequencing:*<br>- HF (n=16)<br>- Control (n=8)<br>*qRT-PCR validation:*<br>- HF 9n=108)<br>- Control (n=35)	miRNA	• The expression of miR-195-3p increased 69.5-fold in the ischemic HF (IHF) group 8.5-fold in the non-ischemic HF (NIHF) group.<br>• In qRT-PCR validation, miR-195-3p expression in the IHF and NIHF groups versus the NC group were significantly increased 2.7975 and 5.2695 fold<br>• In ROC curve analysis, miR-195-3p showed AUC of 0.831 between HF and NC group
Hedayat et al., 2018 [47]; Iran	• Peripheral blood;<br>- HF (n=43)<br>- healthy control (n=140)	SNP	• TNF-α A/A genotype at position -238 was increased in HF patients when compared to controls (OR, 10.20; 95% CI, 1.03-100.8; p=0.043)<br>• TNF-α G/A genotype at position -238 was decreased in HF patients when compared to controls (OR, 0.37; 95% CI, 0.17-0.83; p=0.018)<br>• The most frequent haplotype for TNF-α was A/A in HF patients in comparison with controls (OR, 29.95, 95% CI, 1.6-562.4; p=0.003)

Hua et al., 2020 [23]; China	• Myocardial tissue;<br>- HF (n=21)<br>- control (n=9)<br><br>• Peripheral blood;<br>- HF (n=139)	Transcriptome, miRNA, lncRNA	• 4 fibrosis-associated genes were identified to be significantly related with survival rate: ASPN, COL1A1, COLQ and IGFBP3<br>• COL1A1 showed a negative correlation with survival status: $p=6.1 \times 10^{-3}$ for initial symptoms to heart transplantation and p=0.04 for HF onset to heart transplantation<br>• Immunohistochemical staining assay revealed that the proportion of COL1A1-positive area in HF was much larger than normal control (13.61 ± 2.55% vs 3.76 ± 0.64%, $p=1.1 \times 10^{-3}$<br>• Plasma COL1A1 content greater than 256.5 ng/ml was associated with poor survival within 1 year (HR:7.4, 95% CI, 3.5-15.8, $p<1.0 \times 10^{-4}$
Kao et al., 2017 [42]; USA	• Peripheral blood;<br>- HFpEF (n=284)<br>- Control (n=2754)	SNP	• The rs6696224 (minor allele frequency 11.2%) was associated with all and incident HFpEF (OR 7.6 and 11.3, respectively)<br>• rs5871 (OR 11.7), rs2466052 (OR 10.1) and rs10759715 (OR 4.6) were significantly associated with HFpEF<br>• Two SNPs were significantly associated with HFpEF in the absence of specific risk factors: rs6696224 (AF, CAD, COPD, HTN) and rs3823879 (DM)<br>• Three SNPs had a suggestive association with HFpEF in the absence of specific risk factors: rs604983 (CKD), rs2762941 (COPD), rs882520 (DM)<br>• SNPs significantly associated with HFpEF were related to TGFBR3 (rs6696224), NRG1 (rs2466052), and ELN (rs3823879). Suggestive SNPs were related to CALM1

			(rs5871), BCL9 (rs604983), CYP24A1 (rs2762941), and ARHGEF1 (rs882520)
Lai et al., 2015 [71]; Hong Kong	• Peripheral blood;<br>- HF (n=17)<br>- control (n=17)	Transcriptome, miRNA	• miR-1, miR-21, miR-23, miR-29, miR-130, miR-195 and miR-199 had significant up-regulation in the patient group when compared with the control (p < 0.05)<br>• mRNA expression of casp3, coll I, coll III and TGF had significant up-regulation (p < 0.05) in the patient group
Li et al., 2015 [24]; China	• Myocardial tissue;<br>- HF (n=14)<br>- control (n=10)<br><br>• Peripheral blood;<br>- HF (n=45)<br>- Control (n=45)	miRNA	• During heart failure, among the 51 cardiac-upregulated miRNAs, 8 miRNAs were upregulated, 13 miRNAs were down-regulated in plasma<br>• 3 out of the 23 cardiac down-regulated miRNAs showed a decreasing trend and 8 miRNAs were upregulated in plasma<br>• 8 of the 12 selected miRNAs (miR-660-3p, miR-665, miR-1285-3p, miR-4491, miR-206, miR-1268b, miR-130-3p and miR-330-3p) were successfully validated in the second cohort<br>• In ROC analysis, miR-660-3p, miR-665, miR-1285-3p and miR-4491 exhibited high accuracy for diagnosis (> 0.9)<br>• miR-660-3p, miR-665 and miR-1285-3p showed significant correlations with LVEF% (P < 0.05)
Li et al., 2017 [25]; China	• Peripheral blood;<br>- HF (n=27)<br>- healthy control (n=20)	DNA methylation	• The DNA methylation level of MPV17L and SLC2A1 were lower in HF group than control, while the

			level of PLEC was higher in HF group than control (not significant)<br>• MPV17L and SLC2A1 had an increasing gene expression in HF, while PLEC had a decreasing gene expression (p<0.001 for SLC2A1, p=0.003 for MPV17L, and p=0.002 for PLEC)<br>• The protein concentrations of SLC2A1, MPV17L and PLEC in leukocytes were 5286.3±2558.2 pg/ml (p=0.007), 376.1±287.8 pg/ml (p=0.04) and 35970.4±17646.3 pg/ml (p=0.03) respectively in HF patients, while the protein levels in controls were 2478.9±614.6 pg/ml, 155.3±64.9 pg/ml and 86773.4±47142.4 pg/ml respectively
Mahmoudi et al., 2014 [48]; Iran	• Peripheral blood;<br>- HF (n=43)<br>- healthy control (n=139)	SNP	• The frequency of the IL-4 –590/T allele in the patient group was significantly higher than in the control group (73.3% in patients vs. 46.4% in controls, p < 0.0001)<br>• The most frequent genotypes in patients with IHF were IL-4 CC genotype at position –590 (51.1% in patients vs. 7.2% in controls, p < 0.0001), IL-4 CC genotype at position –33 (65% in patients vs. 43.9% in controls, p = 0.021), and IL-4 TT genotype at position –33 (15% in patients vs. 0% in controls, p < 0.0001)<br>• IL-4 TG at position –1098 (39.6% in patients vs. 59% in controls, p = 0.035), IL-4 TC at position –590 (44.2% in patients vs. 92.8% in controls, p < 0.0001), and IL-4 TC at position –33 (20% in patients vs. 56.1% in controls, p < 0.0001) all

			showed significantly lower frequency in HF group<br>• The most frequent haplotype in our patients was IL-4 TCC which was significantly higher than in the control group (47.5% in patients vs. 23.4% in controls, $p < 0.0001$)<br>• IL-4 TCT (5% in patients vs. 0.7% in controls, $p = 0.0242$) and GCT (3.75% in patients vs. 0% in controls, $p = 0.0108$) haplotypes were significantly more common in the patient group<br>• IL-4 GCC (17.5% in patients vs. 30% in controls, $p = 0.032$), TTT (15% in patients vs. 27.3% in controls, $p = 0.0268$), and TTC (8.75% in patients vs. 18.3% in controls, $p = 0.0399$) showed significantly lower frequency in HF group
Mahmoudi et al., 2016 [49]; Iran	• Peripheral blood;<br>- HF (n=43)<br>- healthy control (n=140)	SNP	• A significant positive association for IL-1β -511/C allele (69 % vs 55.4 %, $p = 0.031$) with ischemic heart failure was found<br>• IL-1β C/C genotype at position -511 was significantly overrepresented in patients with ischemic heart failure compared to healthy controls (45.2 % vs 25.8 %, $p = 0.022$)

Mahmoudi et al., 2018 [50]; Iran	• Peripheral blood;<br>- HF (n=56)<br>- healthy control (n=139)	SNP	• IL-2 -330 G/G and IL-2 +166 T/T genotype was increased in HF patients when compared to controls (OR, 3.56; 95% CI, 1.32-9.57; p=0.013 and OR, 6.72; 95% CI, 1.26-35.71; p=0.022, resp.)<br>• IL-2 -330 G/T genotype was decreased in HF patients when compared to controls (OR, 0.51; 95% CI, 0.27-0.97; p=0.049
Mahmoudi et al., 2019 [51]; Iran	• Peripheral blood;<br>- CHF (n=57)<br>- healthy control (n=140)	SNP	• Higher frequency of heterozygous GC in TGF-β1 at codon 25 in controls compared to CHF cases (12.3% in controls versus 2.2% in patients, P=0·047)<br>• heterozygous CT in TGF-β1 at codon 10 was found to be more frequent in healthy controls compared to patients with CHF<br>• the prevalence of homozygous CC in TGF-β1 at codon 10 was lower in controls than in patients (14.5% in controls versus 32% in patients, P=0·011)<br>• IL-10 ATA haplotype at positions -1082, -819 and -592 was found to be more frequent in healthy controls in comparison with patients group (28.9% in controls versus 15.2% in patients, P = 0·004)<br>• a positive association was detected between TGF-β1 CG haplotype at codon 10 and codon 25 and individual susceptibility to CHF (56.7% in patients versus 39.9% in controls, P=0·007)<br>• TGF-β1 TG haplotype at codon 10 and codon 25 was significantly lower

			than controls (40% in patients versus 52.5% in controls, P=0·04)
Marques et al., 2016 [65]; Australia	• Aortic and coronary sinus blood;<br>- HF (n=9)<br>- Healthy control (n=8)	miRNA	• miR-222-3p was differentially expressed in arterial samples (controls 0.85 vs. patients 1.93, fold change 2.27, P = 0.033)<br>• miR-16, miR-27a-3p, miR-27b-3p, miR-29b-3p, miR-29c-3p, miR-30e, miR-92a-3p, miR-125b, miR-140, miR-195, miR-424, and miR-451a were significantly down-regulated in coronary sinus blood of heart failure patients<br>• let-7a, let-7c, let-7e, miR-23b-3p, miR-107, miR-155, miR-181a, miR-181b, and miR-320a were up-regulated in coronary sinus blood of heart failure patients<br>• PCWP was negatively correlated with miR-16 (r = −0.49, P = 0.048), miR-195 (r = −0.57, P = 0.017), miR-29b-3p (r = −0.63, P = 0.001), miR-29c-3p (r = −0.53, P = 0.030), miR-451a (r = −0.64, P = 0.006), and miR-92a-3p (r = −0.59, P = 0.014)<br>• LV mass was negatively correlated with miR-451a (r = −0.55, P = 0.042)<br>• In ROC analysis, miR-29b-3p, miR-29c-3p, and miR-451a showed highest AUC (all 0.875)<br>• The failing heart released let-7b-5p, let-7c-5p, let-7e-5p, miR-122-5p, and miR-21-5p, and absorbed miR-16-5p, miR-17-5p, miR-27a-3p, miR-30a-5p, miR-30d-5p, miR-30e-5p, miR-130a-3p, miR-140-5p, miR-199a-5p, and miR-451a

Meder et al., 2017 [60]; Germany	• Peripheral blood;<br>- DCM (n=41)<br>- healthy control (n=31)<br>• Cardiac tissue;<br>- DCM (n=41)<br>- healthy control (n=31)	DNA methylation	• 59 CpGs to be significantly differentially methylated in the myocardium of patients with DCM (FDR corrected P≤0.05), with 30 sites that were hypomethylated and 29 sites hypermethylated in DCM<br>• 3 epigenome-wide significant loci were found: cg16318181, $P=2.3\times10^{-8}$; cg01977762, $P=2.8\times10^{-8}$; cg23296652, $P=4.8\times10^{-8}$)<br>• Three epigenetic loci significantly overlapped between tissue and blood (OR, 28; Fisher exact P<0.001) with resolved genes B9 protein domain 1 (B9D1, hypomethylated in DCM in heart tissue and blood), doublecortin-like kinase 2 (hypomethylated), and neurotrimin (hypermethylated)
Ovchinnikova et al., 2016 [56]; The Netherlands	• Peripheral blood:<br>*Discovery phase:*<br>- Advanced HF (n=10)<br>- CHF (n=10)<br>- Control (n=10)<br>*Extended cohort:*<br>- Advanced HF (n=100)<br>- CHF (n=10)<br>- Control (n=14)<br>*Validation cohort:*<br>- Advanced HF (n=9)<br>- CHF (n=10)<br>- COPD (n=8)<br>- Control (n=17)	miRNA	• 40 miRNAs significantly different in AHF patients compared with healthy controls<br>• 15 miRNAs with advanced HF (AHF) showed a highly consistent pattern of decreased miRNA levels with increased acuity of HF (P < 0.001)<br>• In the AHF validation cohort, the levels of seven miRNAs (let-7i-5p, miR-18a-5p, miR-18b-5p, miR-223-3p, miR-301a-3p, miR-423-5p miR-652-3p) were significantly lower in AHF patients compared with healthy controls<br>• After admission, a further decrease in these seven miRNA levels after 48 h was found to be predictive for 180-day mortality

Parsa et al., 2011 [43]; USA	• Peripheral blood;<br>- HF (n=1610)<br>- control (n=463)	SNP	• SNP rs2207418 was associated with heart failure (p= 8 × 10−6), RR = 1.85(1.25–2.73, p= 0.0019)<br>• In heart failure, the homozygous minor allele for this SNP was associated with mortality with a hazard ratio of 1.57 (95% CI = 1.25 to 1.97, p= 0.00013) in the unadjusted model, and when adjusted for age and sex this ratio for the homozygous minor allele was virtually the same, being 1.51 (95% CI = 1.2 to 1.9, p= 0.0004)
Ramachadran et al., 2017 [44]; USA	• Peripheral blood;<br>- HF (n=22)<br>- control (n=49)	miRNA	• A dose-dependent inverse relationship was observed between Ross score and miR129-5p<br>• ROC curve analysis of miR129-5p data exceeded threshold of ≥75% area contained by the curve (c-statistic 98%, P<0.0001)<br>• A decrease in relative expression of miR129-5p to ≤0.58 predicts the presence of clinical HF with 85% sensitivity and 100% specificity<br>• In samples obtained from 3 individuals at different times during their disease course , serial measurements showed that miR129-5p level decreased with increasing Ross score

Sandip et al., 2016 [26]; China	• Peripheral blood;<br>- HF (n=1713)<br>- healthy control (n=1713)	SNP	After adjustment for multiple cardiovascular risk factors including age, sex, smoking status, diabetes, hypertension, and dyslipidemia:<br>• rs8193037 in IL17A was associated with the risk of congestive heart failure (OR 0.76; 95% CI 0.63–0.90, adjusted P = 0.002)<br>• The association was evident in both ischemic and nonischemic heart failure (P = 0.005 and P = 0.05, respectively)<br>After adjustment for multiple cardiovascular risk factors including age, sex, BMI, smoking status, diabetes, hypertension, dyslipidemia, NYHA functional class and LVEF:<br>• rs4819554 in IL17RA was significantly associated with cardiovascular mortality (hazard ratio [HR] = 1.28; 95% CI = 1.02–1.59, adjusted P = 0.03) after prospective follow-up of 12.7 months
Schiano et al., 2017 [54]; Italy	• Myocardial tissue;<br>- HF (n=4)<br>- control (n=4)	Transcriptome, lncRNA	• ADAMTS8 and ADAMTSL4 were significantly down-regulated in HF compared to healthy controls (-4.54 fold and -2.62 fold, resp.)<br>• 27 lncRNAs were differentially expressed in HF vs normal controls (24 up-regulated and 3 down-regulated)<br>• RNA-seq analysis revealed the differential expression of 5 genes encoding the subunits of the mediator complex (MED): MED12, MED13L, MED14, MED17 and MED23

Schneider et al., 2017 [45]; USA	• Peripheral blood<br>*E5103 cohort:*<br>- CHF (n=68)<br>- Control (n=987)<br>*E1199 cohort:*<br>- CHF (n=47)<br>- Control (n=883)<br>*BEATRICE cohort:*<br>- CHF (n=24)<br>- Control (n=298)	SNP	• Nine independent chromosomal regions represented by eleven top SNPs were associated with the risk of CHF (p-value <10−5)<br>• rs28714259 that demonstrated a borderline increased CHF risk (p=0.04, OR=1.9)<br>• rs28714259 was significantly associated with a decreased left ventricular ejection fraction (p=0.018, OR=4.2)
Scrutinio et al., 2017 [55]; Italy	• Peripheral blood;<br>*Screening phase:*<br>- mild to moderate HF (n=5)<br>- advanced HF (n=5)<br>- healthy control (n=5)<br>*Validation phase:*<br>- mild to moderate HF (n=25)<br>- advanced HF (n=29)<br>- healthy control (n=15)	miRNA	• In screening phase, miR-26a-5p, miR-150-5p, miR-485-3p, miR-487b-3p were downregulated while miR-145-3p was upregulated in advanced HF when compared to healthy controls and mild to moderate HF<br>• In validation phase, miR-150-5p was significantly downregulated in advanced HF compared with both healthy control (-1.6 - -3.4 fold) and mild to moderate HF (-1.6 - -2.3 fold).<br>• miR-150-5p was associated with HF severity:<br>- correlated with left ventricular ejection fraction (r=0.43, p=0.001)<br>- correlated with sodium (r=0.29, p=0.029)<br>- correlated with log N-terminal pro-brain natriuretic peptide (r=-0.48, p=0.0002)

			- correlated with left ventricular end-diastolic volume (r=-0.32, p=0.019)<br>- correlated with left ventricular end-systolic volume (r=-0.37, p=0.006)<br>- correlated with bilirubin levels (r=-0.33, p=0.014)<br>- correlated with daily dose of furosemide (r=-0.33, p=0.019)<br>• On univariable Cox analysis, log miR-150-5p expression <–2.2 was associated with a higher risk of the composite end-point of urgent ventricular assist device (VAD) implantation, urgent HT or death (HR = 6.76 [95% CI 1.36 to 33.65]; p = 0.020).
Shah et al., 2020 [72]; UK	• GWAS analysis<br>*Population cohort:*<br>- HF (n=38780)<br>- Control (n=893,657)<br>*Case-control sample:*<br>- HF (n=8529)<br>- Control (n=36,357)	SNP	• 12 independent variants, at 11 loci, associated with HF was found at the genome-wide significance level ($P < 5 \times 10^{-8}$) in the meta-analysis of 29 studies<br>• Six sentinel variants were associated with CAD. Four variants were associated with atrial fibrillation (AF) and two with reduced LV systolic function<br>• A missense variant in BAG3 (rs2234962; $r^2 = 0.99$ with sentinel variant rs17617337) and three missense variants in SYNPO2L (rs34163229, rs3812629 and rs60632610; all $r^2 > 0.9$ with sentinel variant rs4746140) had deleterious effects<br>• Three of 12 variants were significantly associated with the expression of one or more genes located in cis in at least one heart tissue (Bonferroni-corrected $P < 0.05$

			• functional analysis of non-CAD-associated loci implicate genes involved in cardiac development (MYOZ1, SYNPO2L), protein homoeostasis (BAG3), and cellular senescence (CDKN1A)
Sun et al., 2020 [27]; China	• Peripheral blood;<br>- HF (n=30)<br>- control (n=30)	CircRNA	• 696 circRNAs were differentially expressed, 477 were upregulated, and 219 were downregulated in patients with HF<br>• The expression levels of has_circ_0112085 (p = 0.0032), hsa_circ_0062960 (p = 0.0006), hsa_circ_0053919 (p = 0.0074), and hsa_circ_0014010 (p = 0.025) were significantly higher in the HF group than in the controls<br>• The AUCs of hsa_circ_0062960, hsa_circ_0112085, and hsa_circ_0053919 for HF diagnosis were 0.838 ((0.740–0.937), p < 0.0001), 0.817 ((0.713–0.921), p < 0.0001), and 0.759 ((0.631–0.887), p = 0.001), respectively<br>• The serum BNP level was strongly correlated with the expression of hsa_circ_0062960 (R = 0.649, p = 0.003)

Thum et al., 2007 [61]; Germany	• Cardiac tissue;<br>- HF (n=6)<br>- normal adult (n=4)<br>- fetal heart (n=6)	miRNA, transcriptome	• Transcriptome analysis showed a closely related gene expression profile of regulated genes in fetal human hearts compared with failing hearts.<br>• A very close relationship of miRNA expression between fetal and failing hearts was shown. 86.6% of induced miRNAs (>1.5-fold, p<0.05) and 83.7% of repressed miRNAs (>1.5-fold, p<0.05) were regulated in the same direction in fetal and failing heart tissue compared with healthy control.<br>• Upregulated genes had significantly more binding sites for miRNAs repressed in heart failure than downregulated genes (30.2% vs 14.3%, p=0.0002)
Tijsen et al., 2010 [57]; The Netherlands	• Peripheral blood<br>*Cohort 1:*<br>- HF (n=12)<br>- Control (n=12)<br>*Cohort 2:*<br>- Dyspnea registry with HF (30)<br>- Dyspnea registry without HF (20)<br>- Control (n=39)	miRNA	• MiR423-5p distinguished HF cases from healthy controls with an area under the curve (AUC) of 0.91 (95% confidence interval, 0.84 to 0.98)<br>• miR423-5p was highly predictive within the dyspnea registry, when comparing HF and non-HF cases (AUC, 0.83; 95% confidence interval, 0.71 to 0.94).<br>• Circulating miR423-5p correlated with NT-proBNP and EF (Spearman correlation coefficient: 0.43, probability value: 0.002; Spearman correlation coefficient: −0.34, probability value: 0.023, respectively)

Tzimas et al., 2019 [46]; USA	• Cardiac tissue;<br>- Advanced HF with RVF (n=5)<br>- Advanced HF without RVF (n=5)<br>- Healthy donor (n=5)	Transcriptome	• WIPI1 was differentially expressed in RV of BiV-HF hearts versus the RV of either LVF or NF hearts<br>• The expression of WIPI1 correlated with multiple RVF-associated hemodynamic indices
Vegter et al., 2016 [58]; The Netherlands	• Peripheral blood;<br>- acute HF (n=100)<br>- healthy control (n=24)	miRNA	• 7 miRNAs were significantly negatively correlated to biomarkers indicative for a worse clinical outcome in the patient group:<br>- miR-16-5p was correlated to C-reactive protein (R = − 0.66, p-value = 0.0027)<br>- miR-106a-5p was correlated to creatinine (R = − 0.68, p-value = 0.002)<br>- miR-223-3p was correlated to growth differentiation factor 15 (R = − 0.69, p-value = 0.0015)<br>- miR-652-3p was correlated to soluble ST-2 (R = − 0.77, p-value < 0.001)<br>- miR-199a-3p was correlated to procalcitonin (R = − 0.72, p-value < 0.001) and galectin-3 (R = − 0.73, p-value < 0.001)<br>- miR-18a-5p was correlated to procalcitonin (R = − 0.68, p-value = 0.002)<br>• Multiple miRNAs and corresponding predicted target genes were involved in top-enriched pathways, including the PI3K-Akt signaling pathway (p = $1.80e^{-12}$), ErB signaling pathway (p = $6.22e^{-12}$), transforming growth factor–beta (TGF-β) signaling pathway (p = $6.87e^{-11}$) and ubiquitin mediated proteolysis (p = $1.45e^{-10}$).

Wang et al., 2016 [28]; China	• Peripheral blood;<br>- HF (n=569)<br>- healthy control (n=682)	SNP	After adjusting for the potential confounding effects of age, sex, BMI, smoking, drinking, hypertension history, and uric acid:<br>• 3 allelic polymorphisms, rs10932374, rs1595064, and rs13003941, were significantly associated with risk of heart failure<br>- A allele of rs10932374 (OR 0.76, 95% CI 0.61-0.95; p=0.017)<br>- G allele of rs1595064 (OR 0.74, 95% CI 0.60-0.92; p=0.007)<br>- T allele of rs13003941 (OR 1.35, 95% CI 1.06-1.72; p=0.015)<br>• The distributions of genotypes analysis showed that after adjustment, the following SNPs were significantly associated with risk of heart failure:<br>- rs10932374 (OR 0.61, 95%CI 0.39-0.97; p=0.037 in recessive model & OR 0.76, 95% CI 0.61-0.95; p=0.017 in additive model)<br>- rs1595064 (OR 0.58, 95%CI 0.40-0.83; p=0.003 in recessive model & OR 0.75, 95% CI 0.61-0.93; p=0.008 in additive model)<br>- rs13003941 (OR 1.52, 95%CI 1.11-2.09; p=0.009 in dominant model & OR 1.35, 95% CI 1.06-1.73; p=0.015 in additive model)<br>- rs1595065 (OR 1.38, 95% CI 1.02-1.87; p=0.04 in dominant model)

Wang et al., 2019 [29]; China	• Peripheral blood;<br>- HF (n=10)<br>- healthy control (n=10)	lncRNA, miRNA, transcriptome	• Nine DELs were upregulated and 28 DELs were downregulated in the GSE77399 dataset<br>• The GSE104150 dataset contained 96 upregulated and 75 downregulated DEMis<br>• There were 1,430 upregulated and 835 downregulated DEMs in the GSE84796 dataset<br>• The associations between the DELs and DEM is revealed 43 miRNA targets of 12 lncRNAs<br>• The levels of hsa-miR-940, GAS5 and Hotair were higher in the patients with HF compared with the controls.<br>• The circulating levels of hsa-miR-26-5p, hsa-miR-8485 and TUG1 were lower in patients with HF compared with the matched controls
Wong et al., 2015 [73]; Singapore	• Peripheral blood<br>*Screening study:*<br>- HF (n=58)<br>- Control (n=28)<br>*Validation:*<br>- HF (n=60)<br>- Control (n=30)	miRNA	• HF vs. controls<br>- miR-1233 and miR-671-5p upregulated in HF<br>- miR-183–3p, miR-190a,<br>- miR-193b-3p, miR-193b-5p, miR-211-5p, and miR-494 down-regualted in HF<br>• HFrEF vs. controls<br>- miR-125a-5p and miR-671-5p upregulated in HFREF<br>- miR-183-3p, miR-193b-3p, miR-211-5p, miR-494, miR-638 down-regualted in HFrEF<br>• HFpEF vs. controls<br>- miR-1233 and -545-5p upregulated in HFPEF<br>- miR-183-3p, miR-190a, miR-193b-3p, miR-193b-5p downregulated in HFpEF<br>• HFrEF vs. HFpEF

			- miR-125a-5p (FC =3.34, P <0.05) was highly upregulated in HFrEF but remained close to control levels in HFpEF<br>- miR-638 (FC = −8.33, P <0.01) was downregulated in HFrEF but remained close to control levels in HFpEF<br>- miR-190a was significantly downregulated in both HFpEF (FC = −3.18, P <0.01) and HF (FC = −2.03, P <0.01) compared with controls<br>- miR-550a-5p showed a directionally opposite expression pattern between HFrEF (FC =1.73, P <0.05) and HFpEF (FC = −1.58, P <0.05)<br>• Combined ROC analyses for miRNA panels strengthened the diagnostic potential by increasing the AUC values to 1.0 (miR-671-5p), 1.0 (miR-671-5p), 0.89 (miR-1233/miR-193b-3p), and 0.80 (miR-190a) for the HF, HFrEF, HFpEF, and HFrEF vs. HFpEF categories, respectively<br>• The combination of miR-125a-5p and NT-proBNP improved the AUC value to 0.91, which was better than using NT-proBNP alone with an AUC of 0.83 to differentiate HFrEF from HFpEF

Wu et al., 2009 [62]; Taiwan	• Peripheral blood;<br>- HF (n=176)<br>- healthy control (n=176)	SNP	• In a single locus analysis, SNP rs16860760, rs389566, and rs5186 were associated with DHF (allele specific P = 0.004, 0.002, 0.002, respectively; permuted P = 0.045, 0.022, 0.027, respectively)<br>• SNP rs389566, with a minor allele frequency of 20.17%, had an odds ratio 2.03 for the autosomal dominant model [AA + AT: TT, 95% confidence interval (CI) 1.29–3.19; P = 0.0012] and 1.73 for the additive model (95% CI 1.21–2.48; P = 0.0018) corresponding to a population attributable risk fraction of 27.21%<br>• The haplotypes in a linkage disequilibrium block of rs389566 (T–A–G and A–A–G) were significantly associated with DHF (permuted P = 0.0125 and 0.0105, respectively).
Wu et al., 2012 [63]; Taiwan	• Peripheral blood;<br>- HF (n=176)<br>- control (n=176)	SNP	• rs2290149 was significantly associated with DHF (nominal P=0.004). The association remained significant after correcting for multiple testing (permuted P=0.031)<br>• The SNP with a minor allele frequency of 9.4%, had an odds ratio 2.14 (95% CI 1.25–3.66; p=0.004) for the additive model and 2.06 for the autosomal dominant model (GG+GA : AA, 95% CI 1.17–3.63; p=0.013)<br>• the haplotype C-C-G-C was associated with an increased risk (OR 2.10 [95% CI 1.53–2.89], permuted P=0.029) of DHF

Wu et al., 2018 [30]; China	• Peripheral blood;<br>- HF (n=28)<br>- healthy control (n=30)	miRNA	• Exo-miR-92b-5p expression was elevated in HFrEF patients compared with control group (p<0.001)<br>• The association of exo-miR-92b-5p and echocardiographic indexes was identified using Spearman correlation analysis.<br>• The log2−delta CT of exo-miR-92b-5p was positively correlated with LAD (r=0.480, P<0.001), LVDD (r=0.434, P=0.001), and LVSD (r=0.429, P=0.001), while it was inversely correlated with LVEF (r=−0.457, P<0.001) and LVFS (r=−0.502, P<0.001). A sensitivity of 71.4% and a specificity of 83.3% were achieved for identifying HFrEF
Xu et al., 2020 [31]; China	• Peripheral blood;<br>- HFpEF (n=62)<br>- HFrEF (n=62)<br>- control (n=62)	miRNA, lncRNA	• miR-30c expression was significantly decreased in HFpEF and HFrEF groups (P value <0.05). The AUC of miR-30c was 0.62<br>• The expression of lncRNA-CASC7 was notably elevated in both plasma (Figure 4B) and peripheral blood monocytes of HFpEF and HFrEF groups (P value <0.05). the AUC for lncRNA-CASC7 was 0.85<br>• The expression of lncRNA-CASC7 and expression of miR-30 was negatively correlated, although no statistical significant was demonstrated

Yan et al., 2020 [32]; China	• Peripheral blood;<br>- Non-ischemic HF (n=70)<br>- Control (n=77)	miRNA	• GEO data set analysis showed that COX-2, miR-4649 and miR-1297 might be biomarkers for non-ischemic HF.<br>• the plasma level of COX-2 in the non-ischemic HF group was significantly higher than that in control group (36.67 ± 11.97ng/ml vs 27.84 ± 12.82 ng/ml, 95% CI, −12.88 to −4.78, p = 0.00)<br>• miR-4649 was significantly increased in the plasma of patients with non-ischemic HF, compared with control subjects (11.03 ± 10.05 vs 1.00 ± 0.79 fold, 95% CI, −12.43 to −7.63; p = 0.00)<br>• Binary logistical regression analysis showed that plasma COX-2 (odds ratio, 1.292; 95% CI, 1.001−1.134; p = 0.047), miR-4649-3p (odds ratio, 3.821; 95% CI, 2.010–7.263; p = 0.000), miR-1297 (odds ratio, 0.031; 95% CI, 0.002–0.514; p = 0.015) were significantly correlated with non-ischemic heart failure

Zakrzewski-Jakubiak et al., 2008 [74]; Canada	• Peripheral blood;<br>- HF (n=58)<br>- Control (n=111)	SNP	• A significant difference of the AGT T235 allele (P = 0.0025) in the HF patients with frequencies of 48.3% in HF patients vs. 31.5% in the controls was found<br>• AGT M174 allele was present in 18.1% of HF patients vs. 10.4% of healthy controls (P = 0.0446)<br>• Haplotype association test showed that AGT M174 and AGT T235 was present in 18.1% of HF patients vs. 8.4% of healthy controls (P = 0.0069)<br>• Exploratory evaluation of gene-gene combinations revealed an indicative association of the AGT (T235)/ACE(D) combined polymorphisms in the HF group (P = 0.02, OR 2.12, 95% CI 1.11, 4.06)
Zhang et al., 2020 [33]; China	• Peripheral blood;<br>- CHF (n=240)<br>- Control (n=240)	SNP, lncRNA	• there were no significant differences in the frequency of genotypes and alleles of the MHRT gene rs3729830, rs76614781, and rs3729828 loci between patients with CHF and the control group (P > .05)<br>• rs7140721: The risk of CHF in the A allele carriers was 1.43 times higher than C allele carriers of the gene (95% CI: 1.23–1.62, P < .001).<br>• rs3729829: The risk of CHF occurrence in subjects carrying AA genotype was 1.79 times higher than the GG genotype carriers (95% CI: 1.30–2.06, P = .001).<br>• there were 4 haploids which were associated with an increased risk of CHF: TAGAACC (adjusted OR = 1.62, 95% CI: 1.04–2.57, P = .03), CAGAATC (adjusted OR = 2.60, 95% CI: 1.82–3.78, P < .01), CAGAATT (adjusted OR = 2.29,

			95% CI: 1.58–3.40, P < .01), and CAGAACC (adjusted OR = 2.27, 95% CI: 1.40–3.44, P < .01)<br>• plasma lncRNA MHRT level in patients with CHF was significantly higher than that in the control group (P < .01)<br>• A significant difference in the mortality of patients with CHF between different genotypes of rs7140721, rs3729829, and rs3729825 loci (P < .001) were observed.<br>• patients with higher plasma lncRNA MHRT had lower PFS, and the difference was significant (P = .02)
Zhang et al., 2021 [34]; China	• Peripheral blood;<br>- HFpEF (n=80)<br>- Control (n=80)	lncRNA	• The levels of TUG1 were significantly enhanced in the observation group in comparison with those in the control group (P < 0.05)<br>• TUG1 levels were significantly augmented in the NYHA III vs. NYHA II group and in the NYHA IV vs. NYHA III group (P < 0.05)<br>• TUG1 levels were positively correlated with the LAD (r=0.881, P<0.05)

Zhang et al., 2021a [35]; China	• Peripheral blood;<br>- HFpEF (n=16)<br>- Control (n=24)	DNA methylation	• The expression of METTL3, METTL4, KIAA1429, FTO, and YTHDF2 was significantly up-regulated in HFpEF patients, compared with healthy controls<br>• WTAP has a decreased trend (Figure 1D), and ALKBH5 has an increased trend (P=0.07)<br>• The expression of METTL14, YTHDF1, YTHDF3, YTHDC1, and YTHDC2 remained unchanged between these two groups<br>• METTL4 was negatively correlated with TC (r = −0.3632, P = 0.0295) and HDL-C (r = −0.4186, P = 0.0111)<br>• KIAA1429 was negatively correlated with TC (r = −0.4137, P = 0.0121) and LDL-C (r = −0.3457, P = 0.0389)<br>• Protein folding, ubiquitin-dependent ERAD pathway, and positive regulation of RNA polymerase II were significantly altered biological processes in HFpEF.

Zheng et al., 2019 [36]; China	• Epicardial adipose tissue;<br>- HF (n=5)<br>- normal group (n=5)	Transcriptome, lncRNA	• 85 lncRNA and 866 mRNA whose levels changed significantly (p<0.05) were identified, including 45 upregulated and 40 downregulated lncRNA, as well as 404 upregulated and 462 downregulated mRNA<br>• Using a 2-fold expression difference as a cutoff, a total of 30 differentially expressed lncRNAs (17 upregulated and 13 downregulated) and 278 differentially expressed mRNAs (129 upregulated and 149 downregulated) were discriminated between CAD patients with and without HF<br>• lncRNA ENST00000610659 was the top upregulated lncRNA with highest fold change and corresponded to UNC93B1 gene<br>• lncRNA ENST00000610659 and UNC93B1 mRNA were both significantly increased in HF patients in qRT-PCR validation (p=0.040 for lncRNA ENST00000610659 and p=0.019 for UNC93B1 mRNA)<br>• For upregulated genes, the top enriched GO terms in three domains were regulation of lymphocyte activation (GO:0051249) in BP, T cell receptor complex (GO:0042101) in CC, and phosphotyrosine residue binding (GO:0001784) in MF; for downregulated genes, those were oxidation reduction process (GO:0055114) in BP, extracellular space (GO:0005615) in CC, and oxidoreductase activity (GO:0016491) in MF, respectively<br>• The top 3 significantly upregulated pathways were T cell receptor signaling pathway (hsa04660), primary immunodeficiency

			(hsa05340), and endometrial cancer (hsa05213). The significantly downregulated pathways were drug metabolism cytochrome P450 (hsa00982), tyrosine metabolism (hsa00350), complement and coagulation cascades (hsa04610), and Jak-STAT signaling pathway (hsa04630).
